# Genetic and biochemical characterization of a radical SAM enzyme required for post-translational glutamine methylation of methyl-coenzyme M reductase

**DOI:** 10.1128/mbio.03546-24

**Published:** 2025-01-08

**Authors:** Roy J. Rodriguez Carrero, Cody T. Lloyd, Janhavi Borkar, Shounak Nath, Liviu M. Mirica, Satish Nair, Squire J. Booker, William Metcalf

**Affiliations:** 1 Department of Microbiology, University of Illinois Urbana-Champaign, Champaign, Illinois, USA; 2 Department of Chemistry, Pennsylvania State University, University Park, Pennsylvania, USA; 3 Department of Biochemistry, University of Illinois Urbana-Champaign, Champaign, Illinois, USA; 4 Department of Chemistry, University of Illinois Urbana-Champaign, Champaign, Illinois, USA; 5 Center for Biophysics and Quantitative Biology, University of Illinois Urbana-Champaign, Champaign, Illinois, USA; 6 Department of Biochemistry and Molecular Biology, Pennsylvania State University, University Park, Pennsylvania, USA; 7 The Howard Hughes Medical Institute, Pennsylvania State University, University Park, Pennsylvania, USA; University of Massachusetts Amherst, Amherst, Massachusetts, USA

**Keywords:** methyl-coenzyme M reductase, methanogenesis, post-translational modification, archaea, *Methanosarcina*

## Abstract

Methyl-coenzyme M reductase (MCR), the key catalyst in the anoxic production and consumption of methane, contains an unusual 2-methylglutamine residue within its active site. *In vitro* data show that a B12-dependent radical SAM (rSAM) enzyme, designated MgmA, is responsible for this post-translational modification (PTM). Here, we show that two different MgmA homologs are able to methylate MCR *in vivo* when expressed in *Methanosarcina acetivorans*, an organism that does not normally possess this PTM. *M. acetivorans* strains expressing MgmA showed small, but significant, reductions in growth rates and yields on methylotrophic substrates. Structural characterization of the Ni(II) form of Gln-methylated *M. acetivorans* MCR revealed no significant differences in the protein fold between the modified and unmodified enzyme; however, the purified enzyme contained the heterodisulfide reaction product, as opposed to the free cofactors found in eight prior *M. acetivorans* MCR structures, suggesting that substrate/product binding is altered in the modified enzyme. Structural characterization of MgmA revealed a fold similar to other B12-dependent rSAMs, with a wide active site cleft capable of binding an McrA peptide in an extended, linear conformation.

**IMPORTANCE:**

Methane plays a key role in the global carbon cycle and is an important driver of climate change. Because MCR is responsible for nearly all biological methane production and most anoxic methane consumption, it plays a major role in setting the atmospheric levels of this important greenhouse gas. Thus, a detailed understanding of this enzyme is critical for the development of methane mitigation strategies.

## INTRODUCTION

Methyl-coenzyme M reductase (MCR) is the central catalyst in the biological methane cycle and plays a key role in climate homeostasis due the potent greenhouse potential of this gas. The enzyme is found solely in methanogenic and methanotrophic archaea, which are ubiquitous microorganisms inhabiting a variety of anoxic environments (1, 2). During methanogenesis, MCR catalyzes the final step of methane production, while it operates in the reverse direction in anoxic methanotrophs, activating methane for subsequent catabolism. Significantly, these reactions comprise key steps of the global carbon cycle, with methanogens producing gigatons of methane annually and anoxic methanotrophs consuming more than 90% of the methane produced in marine sediments (2).

MCR is a highly unusual enzyme, catalyzing the reversible conversion of methyl-coenzyme M (2-methylthioethanesulfonate, methyl-CoM) and coenzyme B (N-7 mercaptoheptanoylthreoninephosphate, CoB) to methane and the heterodisulfide of CoB and CoM (CoM-S-S-CoB). Although the mechanism of this reaction has yet to be fully elucidated, current evidence suggests that heterolytic cleavage of methyl-CoM forms a rare methyl radical intermediate that reacts with coenzyme B to produce methane and a CoB radical that recombines with CoM to form CoM-S-S-CoB (3). This intriguing reaction involves a nickel-containing porphinoid cofactor, known as F_430_, found solely in MCR and the closely related alkyl-CoM reductases (4). For MCR to be catalytically active, the nickel within F_430_ must be in the Ni(I) oxidation state; however, the cofactor is readily oxidized to an inactive Ni(II) state, which has made *in vitro* biochemical characterization of the enzyme extremely challenging (2).

Structural characterization of MCR from *Methanothermobacter marburgensis* showed the enzyme to be an intertwined hexamer with two copies each of three subunits (α_2_/β_2_/γ_2_), and five post-translationally modified amino acids near the active site on the α subunit: 1-N-methylhistidine, 5-methylarginine, S-methylcysteine, 2-methylglutamine, and thioglycine (5). Characterization of additional MCRs revealed several more post-translationally modified residues, including didehydroaspartate, 6-hydroxytryptophan, 7-hydroxytryptophan, methylisoleucine, and 2-N-methylhistidine (6–11). The distribution of these post-translational modifications (PTMs) varies between species, with some being widely conserved and others being seen only rarely (12). Despite these variations, all characterized MCRs possess at least three post-translationally modified amino acids near the active site, suggesting that the PTMs play an important role in either catalysis or protein stability (2, 12, 13). Although experimental evidence regarding the function of these PTMs is scarce, considerable progress has been made in identifying the genes and enzymes required for these modifications.

The enzyme that converts glycine to thioglycine was identified as a homolog of ones that catalyze similar sulfur chemistry during synthesis of thiazole natural products (14). The encoding gene, designated *ycaO*, is often found adjacent to the MCR operon, providing further support for this proposal. *Methanosarcina acetivorans* mutants with deletions of *ycaO* produce MCR without thioglycine, establishing its role in synthesis of this PTM (14). In addition, three different YcaO homologs have been shown to covert glycine to thioglycine *in vitro* (15).

Two types of enzymes are involved in post-translational methylations of MCR. Simple nucleophilic substitutions by canonical SAM-dependent methyltransferases are routinely used for N and S methylations, like those found in S-methylcysteine and N-methylhistidine. Consistent with this idea, the canonical SAM-dependent methyltransferase encoded by the *mcmA* gene is responsible for the S-methylcysteine modification, based on absence of this PTM in *M. acetivorans mcmA* deletion mutants (16). In contrast, methylation at the C2 position of glutamine, or the C5 position of arginine, requires stronger chemistry, such as that used by radical SAM (rSAM) enzymes (17). In particular, the B12-dependent subfamily of rSAMs commonly catalyze transfer of methyl groups to *sp*^3^-hybridized carbons atoms like those in 2-methylglutamine and 5-methylarginine PTMs (18). The *mamA* gene (also known *mmp10* or *mmpX*) encodes a member of this family that is responsible for 5-methylarginine synthesis as shown by the failure of *M. acetivorans*, and *Methanococcus maripaludis mamA* mutants make this PTM, and the ability of purified MamA is able to methylate peptide substrate mimics (16, 19, 20). The structure, substrate specificity and redox characteristics of *M. acetivorans* MamA have recently been determined, providing the clearest picture to date of the enzymatic mechanism for members of the B12-dependent rSAM family (21). Finally, during the course of the work described here, the Layer group reported *in vitro* activity of MgmA from *Methanoculleus thermophilus*, a B12-dependent rSAM methyltransferase responsible for the 2-methylglutamine PTM (22). *In vivo* characterization of *mgmA* mutants has yet to be reported.

Here, we show that MgmA homologs can methylate the target glutamine in McrA *in vivo* when heterologously expressed in *M. acetivorans*, which does not normally contain this PTM. Recombinant strains that express MgmA show small, but significant, grow defects on methylotrophic substrates showing that the methylglutamine PTM effects MCR activity, a finding that is supported by the observation of altered cofactor binding in the crystal structure of Gln-modified MCR. Structural studies of MgmA reveal a canonical domain B12-dependent rSAM methyltransferase with a large active site suitable for binding unfolded MCR.

## RESULTS

### Bioinformatic identification of genes involved in PTM of MCR

**Fig 1:**
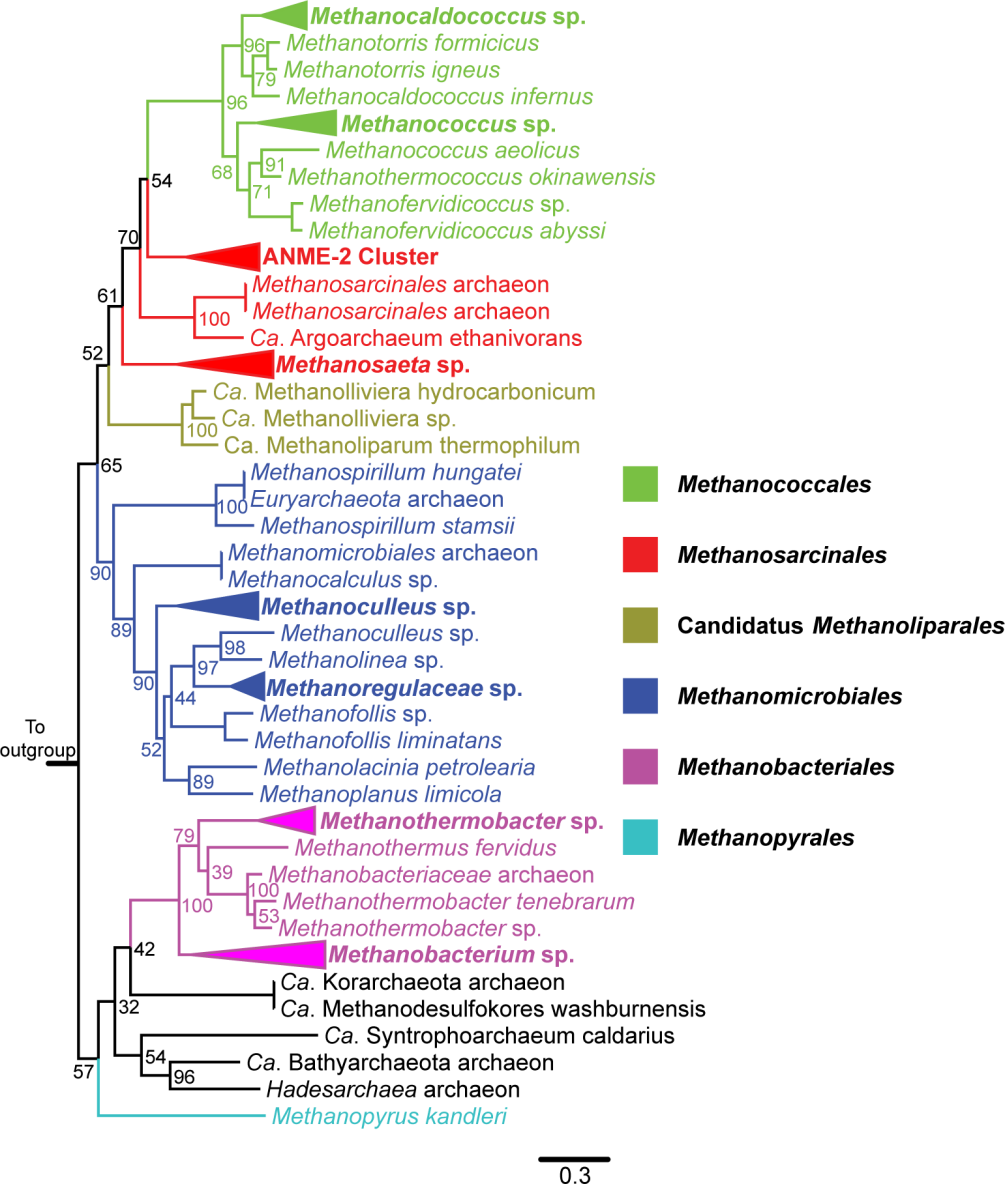
The phylogeny of MgmA homologs. A maximum-likelihood phylogenetic tree of MgmA homologs is shown. Branches are color-coded based on the archaeal order of the organisms in which each homolog is found, as indicated by the key within the figure. The node labels indicate bootstrap support of 100 resamples. MiaB, an rSAM protein responsible for tRNA modification in bacteria, was used as an outgroup used to root the tree.

To identify genes that could be invovled in post-translational glutamine methylation, we compared the genomes of *M. marburgensis*, *Methanopyrus kandleri*, and *Methanotorris formicicus*, which have this PTM, to those of *M. acetivorans* and *Methanosarcina barkeri*, which do not. This produced a list of 77 candidates that are present only in strains with this PTM (Table S1). Among these, only two encode rSAM methyltransferases. The first is the *hcgA* family, which is involved in biosynthesis of novel hydrogenase cofactor (23). The second, which we designated *mgmA*, encodes a family of putative B12-dependent rSAM methyltransferases. MgmA homologs are encoded by organisms within every euryarchaeal order that includes methanogens except the *Methanomassiliicoccales* (Fig. 1). Several recently discovered organisms from the TACK superphylum also encode MgmA, as well as MCR and/or the related family of alkyl-CoM reductases (2). Consistent with the idea that *mgmA* plays a role in MCR maturation, we observed the gene to be clustered with the MCR operon in most members of the genera *Methanobacteriales* and *Methanosaeta*. Thus, as previously suggested, MgmA is a strong candidate for synthesis of the methylglutamine residue in MCR (22, 24).

### Expression of MgmA in *M. acetivorans* results in glutamine methylation

**Fig 2:**
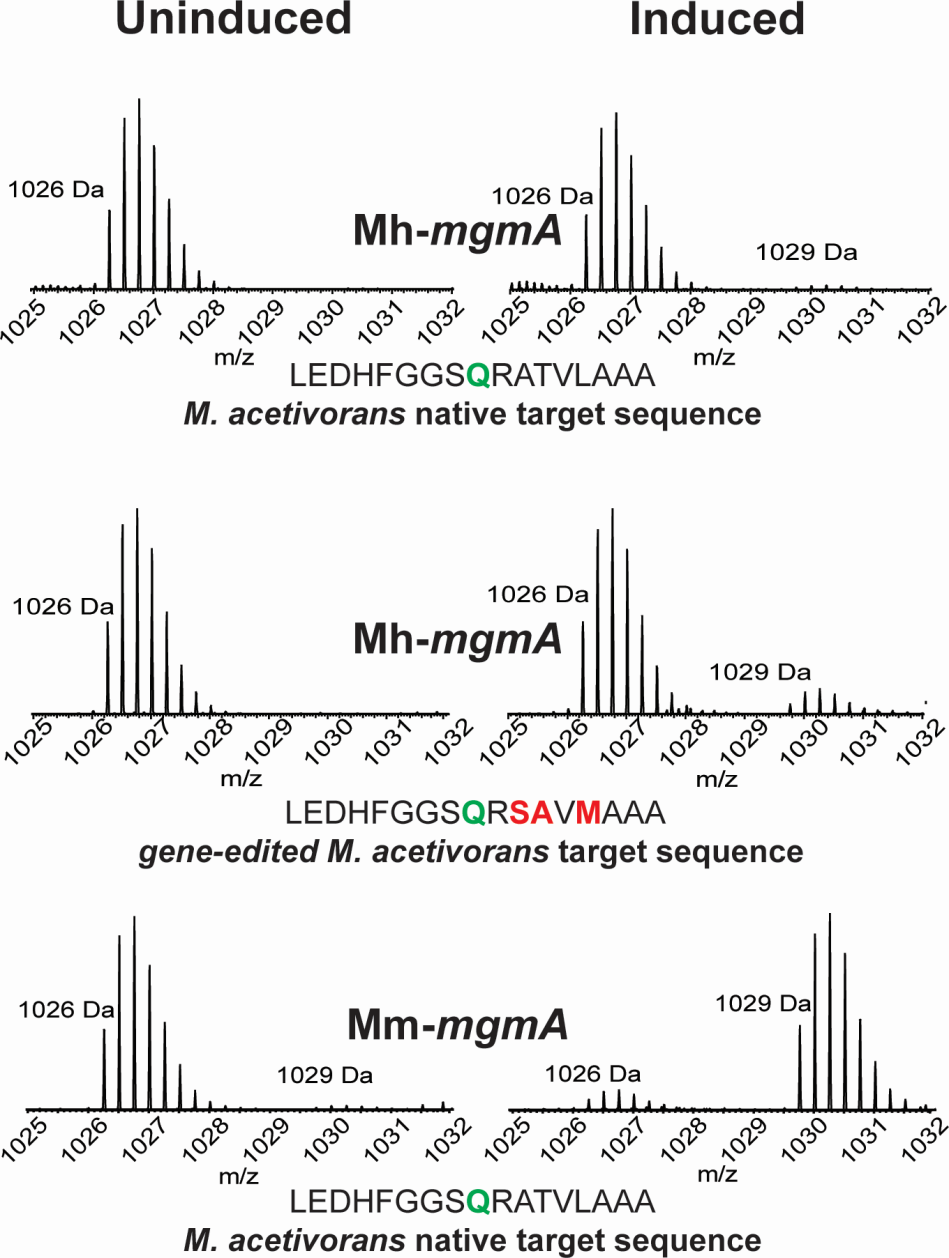
MgmA methylates Gln_420_ of McrA when expressed in *M. acetivorans*. MCR was purified from recombinant *M. acetivorans* strains carrying tightly regulated copies of either Mh-*mgmA* or Mm-*mgmA* after growth under inducing and non-inducing conditions and then subjected to trypsin digestion and mass spectrometric analysis. The ESI-MS spectra of the McrA peptide containing the target glutamine are shown, with uninduced and induced samples on the left and right of each panel, as indicated. The top panel shows the spectrum from an *M. acetivorans* strain expressing Mh-*mmgA*; the middle panel, the spectrum from an Mh-*mmgA*-expressing strain in which McrA, has gene-edited to match the local sequence of *M. harundinacea* expressing Mh-*mmgA*; the bottom panel shows the spectrum from an Mm-*mmgA-*expressing strain. The target sequence is shown for each panel, with the target residue in green and mutated residues in red. The series of peaks beginning at *m*/*z* of 1,026.2622 (*z* = 4) are consistent with an unmethylated peptide, while those beginning at *m*/*z* of 1,029.7663 (*z* = 4) are consistent with a single methylation. MS/MS analysis of the methylated peptides confirms the identity of the indicated peaks, as well as localizing the mass shift to a 2–4 amino acid region containing Gln_420_ (Fig. S1 to S3).

To show that MgmA is responsible for the 2-methylglutamine PTM, we expressed the gene from two different methanogens in *M. acetivorans* (14). The gene from the *Methanosaeta harundinacea* was chosen in the hope that the close phylogeny of the two organisms would minimize potential heterologous expression issues; however, because this PTM has not been confirmed in *M. harundinacea*, we also expressed the *mgmA* from *M. marburgensis,* where it is known to occur (5, 25). Each gene was placed under the control of a tightly regulated promoter and inserted into the *M. acetivorans* chromosome in single copy. After growth of these strains with and without inducer, MCR was analyzed via electrospray ionization mass spectrometry (ESI-MS/MS) to establish the MCR methylation state of the peptide containing the Gln_420_ target residue (Fig. 2; Fig. S1 and S2). When the *M. harundinacea* protein (*Mh*MgmA) was expressed, approximately 2% of the target peptide had a mass 14 Da heavier, consistent with a single methylation event. MS/MS analysis of the heavier peptide localized the modification to a 4-amino acid region containing Gln_420_. Thus, *Mh*MgmA can methylate *M. acetivorans* McrA *in vivo*, albeit poorly. To test whether the poor activity of *Mh*MgmA was due to differences in the target sequence between M. *harundinacea* and *M. acetivorans*, we used Cas9-dependent genome editing to change the McrA target sequence of *M. acetivorans* so that the eight upstream, and nine downstream, amino acids were identical to those in *M. harundinacea* McrA. This led to fivefold increase in the amount of the heavier peptide, showing that the altered target sequence improves enzyme activity, but not enough to achieve full methylation of McrA. In contrast, the strain expressing the *M. marburgensis* protein (*Mm*MgmA) showed nearly complete (*ca*. 95%) modification of the target peptide (Fig. 2; Fig. S3). Thus, *Mm*MgmA is an efficient methylase of *M. acetivorans* McrA, despite several differences between the target peptides of the native organisms. Methylation was not observed when the strains were grown under non-inducing conditions.

### Phenotypic consequences of Gln_420_ methylation in *M. acetivorans*

We measured the doubling time and cell yield of *M. acetivorans* expressing Mm-*mgmA* in methanol and trimethylamine (TMA) media to assess the effect of the modified MCR on growth. To ensure that the phenotypes were due to Gln methylation, rather than the metabolic burden of expressing a foreign protein, we also constructed isogenic strains that express an inactive *mgmA* allele in which the three iron-binding cysteine residues of the essential 4Fe/4S cluster were mutated to alanine. Small, but statistically significant, phenotypic differences were observed between strains expressing the active and inactive alleles of *Mm*MgmA (Table 1). Thus, strains expressing the functional allele grew slightly faster on TMA at all temperatures, but with slightly lower growth yields, whereas they grew slightly slower on methanol, without any impact on yield.

**TABLE 1:** Growth phenotypes of *M. acetivorans* expressing active Mm-MgmA (MgmA^act^) and the inactive Mm-MgmA (MgmA^inact^) on different substrates and temperatures^*a*^

Conditions^*b*^	MgmA^act^ doubling time (hr)	MgmA^inact^ doubling time (hr)	MgmA^act^ max OD_600_	MgmA^inact^ max OD_600_
TMA 30°C	**19.2 ± 0.7**	21.7 ± 1.1	**3.3 ± 0.3**	4.1 ± 0.5
TMA 36°C	**10.6 ± 0.2**	12.7 ± 0.3	**3.2 ± 0.2**	4.1 ± 0.2
TMA 42°C	**11.1 ± 0.9**	13.9 ± 1.3	**3.4 ± 0.4**	4.1 ± 0.2
MeOH 30°C	**20.5 ± 1.5**	17.9 ± 0.5	4.3 ± 0.5	4.6 ± 0.2
MeOH 36°C	**11.8 ± 0.3**	9.5 ± 0.7	4.0 ± 0.3	4.0 ± 0.3
MeOH 42°C	**14.9 ± 1.0**	12.6 ± 1.3	3.4 ± 0.2	3.6 ± 0.2

^*a*^ Values represent the mean and 95% confidence intervals for three biological replicates. Differences with a *P* < 0.05 (calculated using a two-tailed *t*-test assuming equal variances) were considered significant and are highlighted in bold.

^*b*^ TMA, trimethylamine; MeOH, methanol.

### Biochemical and structural characterization of MgmA

**Fig 3:**
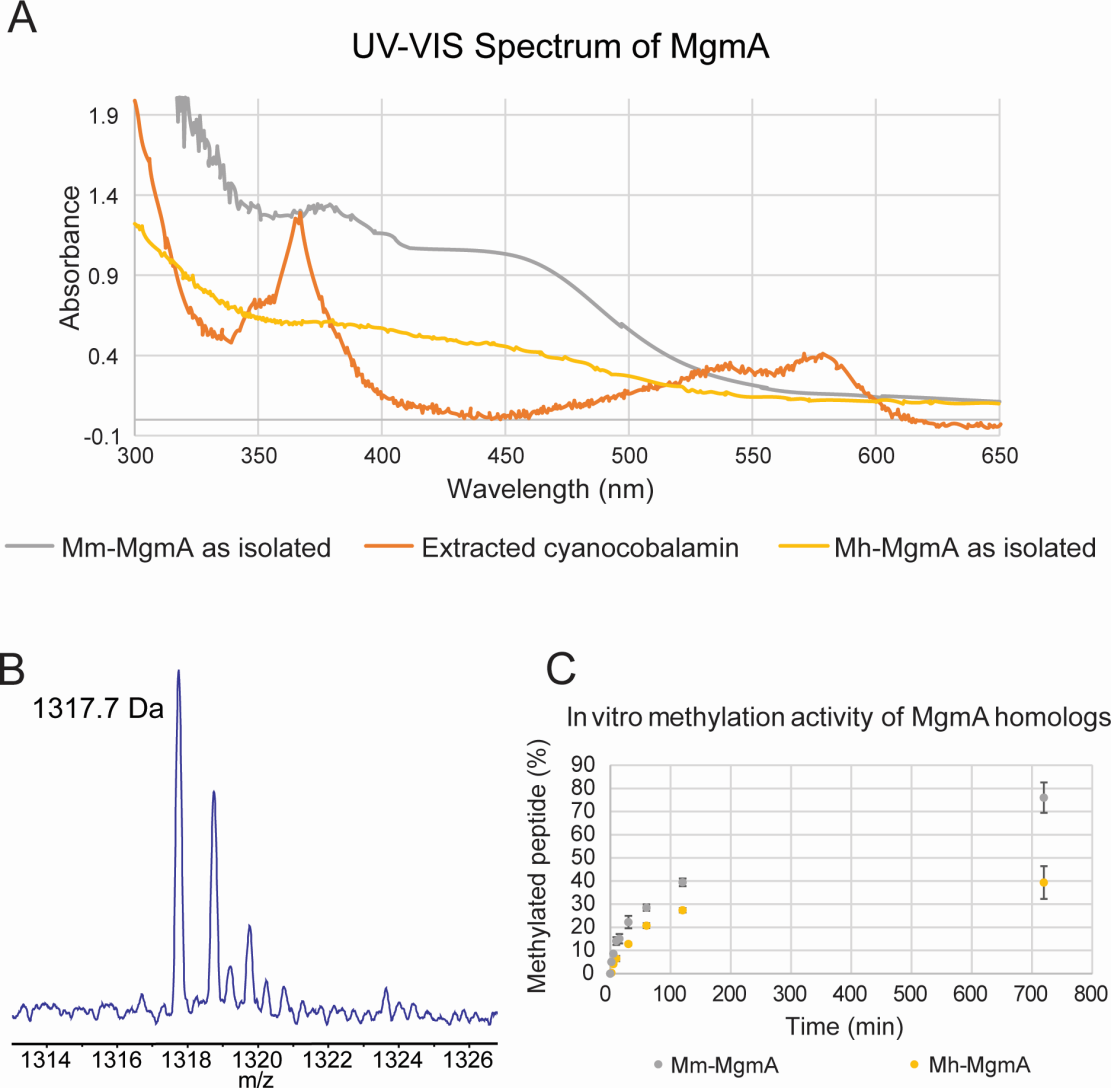
Biochemical characterization of affinity-purified MgmA. (A) UV-vis spectra of as-isolated Mh-MmgA (yellow trace) Mm-MgmA (grey trace), and cyanocobalamin extracted from the Mm-MgmA (orange trace). UV-vis spectra of Mh-MgmA as isolated (yellow). (B) Ion intensity chromatogram of the major peak observed by MALDI-TOF-MS of the cyano-cobamlamine derivative of the cofactor extracted from Mm-MmgA. The cobamide was confirmed to have 5-hydroxybenzimidazolyl as the lower ligand based on the observed mass of 1,317.738 (*m*/*z*) relative to the predicted exact mass of 1,317.535 (*m*/*z*). (C) Methylation activity of Mm-MgmA (yellow trace) and Mm-MgmA (grey trace) using a peptide substrate. The amounts of methylated peptide were determined by MALDI-TOF-MS at the indicated timepoints, with the average and standard deviation of triplicate assays shown.

*Mh*MgmA and *Mm*MgmA were anoxically purified and biochemically characterized after heterologous expression in *M. acetivorans*. The UV-vis spectrum of the as-isolated proteins show a peak at 375 nm with a broad shoulder extending to *ca*. 450 nm, suggesting the presence of intact 4Fe-4S clusters and a cobamide cofactor (Fig. 3). The cofactor was extracted from purified *Mm*MgmA and subjected to matrix-assisted laser desorption ionization-time of flight-MS (MALDI-TOF-MS), which revealed an intact mass consistent with a cobamide containing 5-hydroxybenzimidizole as the lower ligand, which is the native cobamide found in methanogens (22, 26). The *in vitro* methylation activity of the as-purified proteins was assessed via mass spectrometric analysis of a 20-amino acid peptide substrate corresponding to the target region of the *M. acetivorans* McrA. Although *MmMgmA* was significantly more active, both enzymes were able to methylate the peptide substrate, with 76% and 39% of the substrate being consumed in overnight reactions of *MmMgmA* and *MhMgmA*, respectively.

**Fig 4:**
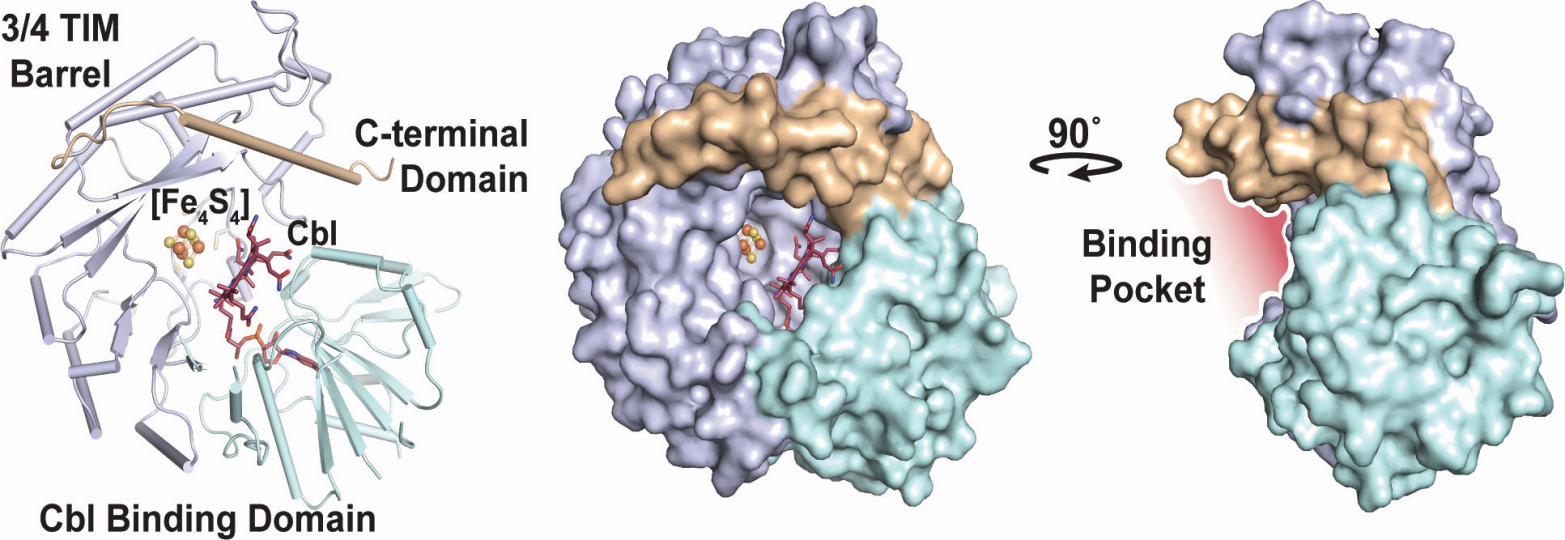
X-ray crystal structure of *Mm*MgmA with bound cofactors. The architecture of *Mm*MgmA showing the Rossman fold (pale cyan) with bound cobalamin (red), ¾ TIM barrel (light blue) with the rSAM [Fe4S4] cluster coordinated by three cysteines, and the C-terminal domain (wheat) is shown on the left. A surface view of *Mm*MgmA revealing the active site opening from the front is shown in the center, with a large peptide binding cleft (red) revealed when the protein is rotated 90 degrees clockwise on the right.

To obtain larger amounts of the enzyme, *Mm*MgmA was expressed in *Escherichia coli,* followed by *in vitro* reconstitution using a commercially available cobamide containing dimethylbenzimidazole as the lower ligand (Fig. S4). Efficient reconstitution was validated by quantification of iron (3.3 +/− 0.4 per protein) and cobalamin (0.89 per protein). The reconstituted enzyme was highly active, with *k*_cat_ values of 0.31 and 0.19 min^−1^ determined by quantification of the reaction products 5′-dAH and SAH, respectively. Subsequent anoxic crystallization allowed determination of a 2.08 Å structure that is similar to other B12-dependent rSAM methyltransferases (Fig. S5, [27, 28]), with a large active site cleft (Fig. 4). Like other B12-dependent rSAM methyltransferases, the benzimidazole tail anchors the Cbl cofactor (dimethybenzimidizole in the reconstituted enzyme) in the active site tucked into the Rossmann fold domain and does not serve as the lower ligand of the cofactor (Fig. S6, [27]). Previously characterized B12-dependent rSAMs have a hydrophobic amino acid in the lower axial position of the Cbl cofactor, which is absent in *Mm*MgmA (28). The closest residue that could serve this function is Gln_49_, which is 8.3 Å away and facing the wrong direction. The lack of a lower cobalt ligand is consistent with electron paramagnetic resonance (EPR) data reported by Gagsteiger et al. (22); however, when rotated around its β-carbon, the amine of Gln_49_ is ideally placed for coordination to the cofactor with a distance of 2.5 Å (Fig. S6), suggesting a possible alternative that may have implications during catalysis.

### Structure and redox state of Gln_420_-methylated MCR from *M. acetivorans* (*Ma*MCR)

**Fig 5:**
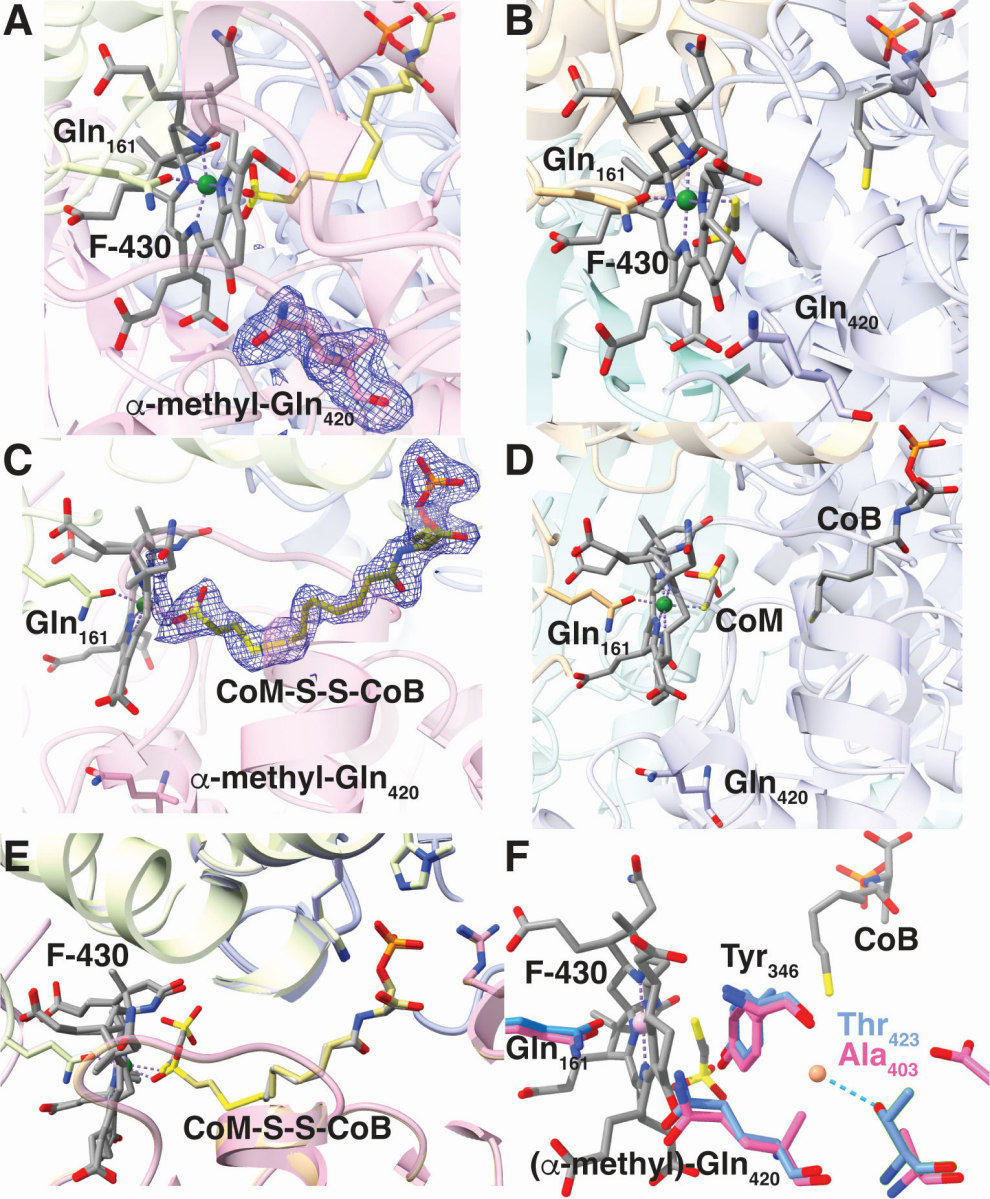
Active site of MaMCR with and without Gln_420_ methylation. (A) The active site of *Ma*MCR purified from a strain expressing *Mm*MgmA, with the outlined electron denisty clearly showing methylation of Gln_420_. (B) The same view as in panel A for unmodified *Ma*MCR. (C) A side view of Gln_420_-methylated *Ma*MCR showing the bound CoM-S-S-CoB heterodisulfide coordinated to the F430 cofactor via the sulfonate moiety of CoM. The position α-methyl-Gln_420_ is also shown. (D) The same view as in panel C for unmodified *Ma*MCR showing the positions of the free CoM and CoB cofactors, as well as the unmodifed Gln_420_. (E) Superposition of the structures of unmodified and modified *Ma*MCR in the vicinity of the CoM and CoB cofactors showing strong conservation of active site features. (F) Superposition of the active sites of unmodified *Ma*MCR and *Mm*MCR (which contains a MeGln residue) showing adaptations that accommodate the methyl group. The water molecule H-bonded to Thr_423_ is shown as an orange sphere.

To assess whether Gln_420_ methylation alters the structure of MCR, we aerobically purified the Ni(II) form of affinity-tagged *Ma*MCR from a strain expressing *Mm*MgmA, allowing determination of structure at 2.0 Å resolution (Fig. 5; Fig. S7). The protein fold seen in the structure is nearly identical to that of the unmodified enzyme with a mean RMSD of 0.25 Å. Clear electron density is seen for α-methylation on Gln_420_, consistent with the studies described above. Surprisingly, the active site contains the CoM-S-S-CoB heterodisulfide positioned near the Ni(II)-F_430_, whereas free CoM and CoB was observed in each of the eight prior MaMCR structures we have obtained (14, 16). The Ni(II) is coordinated by four nitrogen atoms of the tetrapyrrole of F_430_, the side chain oxygen of Gln_161_, and the sulfonate oxygen of the heterodisulfide. Although dozens of MCR structures have been reported (including eight from *M. acetivorans*), the bound heterodisulfide has only been observed once before, in the MCR silent form of the enzyme isolated from of *M. marburgensis* (formerly *Methanobacterium thermoautotrophicum,* PDB Code 1HBM [25]).

To examine whether Gln_420_ methylation affected the redox state of the F_430_ cofactor, modified and unmodified *Ma*MCRs were also isolated under strictly anoxic, highly reducing conditions. The UV-vis spectrum suggests that the majority of the enzyme in both the modified and unmodified preparations is in the MCR-silent Ni(II) state displaying maximum absorbance at 420 nm, with a shoulder at 445 nm (Fig. S8A). Analysis by EPR shows that MCRox1 forms are present in both modified and unmodified *Ma*MCR, with ca. twofold more in the Gln-methylated derivative (Fig. S8B). However, due to data processing issues associated with the strong the Ti-citrate buffer signal, these values have an appreciable systematic error. Thus, we are hesitant to draw strong conclusions from these data.

## DISCUSSION

The data presented here show that MgmA family members methylate the Gln_420_ residue in McrA *in vivo* and are fully consistent with *in vitro* results obtained with the homolog from *M. thermophilus* (22). Interestingly, the MgmA homologs characterized here vary in their methylation activity both *in vivo* and *in vitro*, which is only partially due to differences in the native McrA substrates. This suggests that the enzyme has a limited amount of time to methylate G_420_ before it becomes inaccessible due to protein folding, an idea that is consistent with the large active site cleft of *Mm*MgmA that presumably binds a linear, unfolded peptide substrate.

The key question that has yet to be answered is why some methanogens methylate Gln_420_, while others do not. Based on available MCR structures, the methyl moiety fills a gap that is occupied by water in MCRs without this PTM (11). In *M. barkeri* MCR, this water molecule is part of a hydrogen-bonding network that positions Tyr_346_ for hydrogen bonding with the axial Ni-ligand in F_430_, which is presumably required for efficient catalysis. This key water is held in place via a hydrogen-bond with the nearby Thr_423_ residue, a position that is occupied by Ala in MCRs that have Gln methylation (5). It has been suggested that by filling the gap occupied by this key water molecule, the methyl-Gln PTM also serves to positionTyr_346_ for hydrogen bonding with the axial Ni-ligand (11). Identification of MgmA as the Gln methylase allows us to extend this analysis to a much larger collection of strains. Accordingly, we examined 154 methanogen genomes, which encode 204 McrA homologs due to the fact that some species have two MCR isozymes (Data Set S1). Of these, 62 encode MgmA. Strikingly, 84 of the 85 MCRs found in organisms that encode MgmA have a small hydrophobic residue (always Gly or Ala) in the position analogous to Thr_423_, the only exception being one of the two MCRs encoded by *Methanobacterium formicicum* Mb9. However, when the McrA position analogous to Thr_432_ is occupied by a hydrogen bond donor (always Ser or Thr), the organisms almost never encode MgmA (again the only exception is *M. formicicum* Mb9). These data lend credence to the idea that alternate mechanisms for positioning of Tyr_346_ are important. Nevertheless, it is important to note that the hydrogen-bonding network, including the water molecule in question, remains intact in our Gln-modified *Ma*MCR structure. Thus, the additional methyl group is insufficient to displace water when Thr_423_ is present. It should also be noted that *ca*. one-third of the organisms with Ala or Gly in this position lack MgmA. Therefore, it remains possible that these alternate conformations have distinct functions in organisms with and without methylglutamine. In this regard, it is interesting to note that most of the organisms lacking MgmA are capable of methylotrophic methanogenesis (Data Set S2). Because the vast majority of these methylotrophic methanogens fall within the *Methanosarcinales*, this could be a reflection of phylogeny rather than function, but significant phylogenetic outliers suggest this is not the case. For example, both *Methanobrevibacter* and *Methanospheara* species lack MgmA and are capable of using methanol (29), whereas other genera within the *Methanobacteriales* encode MgmA and are incapable of using methanol. Similarly, members of the methylotrophic orders *Methanomassiliicoccales*, *Methanomethylicales*, and *Methanonatronarchaeales* also lack MgmA. Although the correlation between MgmA absence and methylotrophy is not absolute, these findings suggest that there may be a functional linkage. As we have previously noted (14), methylated substrates are assimilated directly into methyl-CoM, and it is therefore that the cytoplasmic concentration of this substrate differs between methylotrophic, hydrogenotrophic, and aceticlastic methanogens that form methyl-CoM in a different fashion. It is tempting to speculate that the hydrogen-bonding network of methanogens without methylglutamine serves to tune the substrate affinity of the enzyme to higher methyl-CoM concentrations.

Regardless of the function(s) of methyl-Gln and the analogous hydrogen-bonding network, perturbation of this critical region in the active site affects MCR function. This can be seen in two ways. First, phenotypic characterization of *M. acetivorans* strains with and without the 2-methylglutamine PTM revealed statistically significant differences in the use of methylotrophic substrates. While this effect is small, it is important to note that MCR is not the rate-limiting step in the growth of *M. acetivorans*. Indeed, a recent study by the Nayak group showed that MCR expression can be lowered by ~70% before a growth phenotype can be observed (30). Thus, the observation of any phenotype suggests that the enzyme activity has been significantly reduced. Indeed, we cannot exclude the possibility that all growth depends on the small fraction (*ca*. 5%) of unmethylated MCR (see Fig. 2). Second, the Gln modification in *Ma*MCR, which also contains the Thr_423_-dependent hydrogen bonding network, alters the binding of the CoM-S-S-CoB heterodisulfide in way that allows this reaction product to remain bound during enzyme purification, as opposed to the free cofactors seen in each of the eight previous *Ma*MCR structures that we have obtained (14, 16). This suggests that product release has been altered by the modification. Experiments to identify the nature of this perturbation (e.g., effects on K_m_, k_cat_) will await the development of methods to purify the active Ni(I) state of *M. acetivorans* MCR, or the development of efficient genetics for organisms like *M. marburgensis*, from which active MCR can be purified. Unfortunately, neither approach is currently feasible, although recent progress in the development of genetic tools for *M. marburgensis* holds promise for solving this issue (31).

## MATERIALS AND METHODS

### Bioinformatics methods

Standard bioinformatics analyses were performed using webtools available on the National Center for Biotechnolgy Information website. Phylogentic profiling was performed using tools available on the Department of Energy’s Integrated Microbial Genomes website. The protein classification and neighborhood networks were generated using tools of the Enzyme Function Iniative (32).

### Molecular biology methods

Standard molecular biology methods were used throughout. Details of most plasmid constructions are provided in Table S2. Primers and synthetic DNA fragments used in these constructions are listed in Tables S3 and S4. CRISPR gene editing target sequences are listed in Table S5. Plasmid p*Mm*MgmA was used for expression of MmMgmA in *E. coli* and was made by cloning a codon-optimized derivative into pET28a. All plasmids were verified by Sanger sequencing at the Roy J. Carver Biotechnology Center, University of Illinois Urbana-Champaign or the Pennsylvania State Genomics Core Facility.

### Construction and growth of microbial strains

Methods for growth and genetic manipulation of *E. coli* and *M. acetivorans* have been previously described (33–35). *M. acetivorans* strains generated in this project were verified by Sanger sequencing of PCR products containing the modified regions at the Roy J. Carver Biotechnology Center, University of Illinois Urbana-Champaign.

### Purification and mass-spectrometric analysis of *M. acetivorans* MCR

MCR for mass spectrometric analyses of protein modification was purified under aerobic conditions using ammonium sulfate precipitation followed by size exclusion chromatography essentially as described (36). The modification state of MCR was determined by high-resolution electrospray ionization (ESI) MS/MS a Thermo Fisher Scientific Orbitrap Fusion ESI-MS as previously described (14, 16).

### Growth assays for *M. acetivorans* strains

*M. acetivorans* strains were grown in single cell morphology in high-salt medium (37, 38) containing either 125-mM methanol or 50-mM TMA. Growth rate was quantified by measuring the OD at a wavelength of 600 nm using a Spectronic 200E (Thermo Fisher Scientific) spectrophotometer.

### Purification and characterization of MgmA from *M. acetivorans*

TAP-tagged MgmA was purified under anoxic conditions as described (16) with the following changes: lysis buffer was 50-mM HEPES, pH 7.5, 10-mM DTT, wash buffer was 250-mM KCl, and elution buffer was 250-mM KCl with 50-mM biotin. The cobamide cofactor was extracted and identified by mass-spectrometric analysis as previously described (39). MgmA activity assays were performed in triplicate under strictly anoxic conditions at 25°C. The reaction mixture was composed of 20-mM HEPES (pH7.5), 150-µM peptide substrate and 2-mM Ti(III) citrate, 7.5-µM MgmA. The reaction was initiated by addition of SAM to a final concentration of 500-µM. The reaction was quenched by adding an equal volume of 100-mM H_2_SO_4_ and then analyzed via MALDI-TOF-MS using alpha-cyano-4-hydroxycinnamic acid (CHCA) and matrix and a Bruker UltrafleXtreme Mass Spectrometer (Bruker Daltonics, Billerica, MA, USA) in a reflector-positive mode at the University of Illinois School of Chemical Sciences Mass Spectrometry Laboratory. Peptide concentrations were calculated by measuring the ratio between the modified and unmodified peptides (40).

### Purification and characterization of MgmA from *E. coli*

Expression, purification, and reconstitution of *Mm*MgmA were modified from previously established methods after heterologous expression in *E. coli* BL-21(DE3)/pDB1282/pBAD42-BtuCEDF/p*Mm*MgmA (41, 42). Purified protein was designated “as isolated *Mm*MgmA (AI *Mm*MgmA)” prior to reconstitution and “reconstituted *Mm*MgmA (RCN *Mm*MgmA)” following the restoration of the 4Fe-4S cluster and insertion of the cobamide. Fe content of the reconstituted protein was determined as previously described (43). Activity assays contained 20-µM RCN *Mm*MgmA, 200-µM peptide substrate, 500-µM SAM, 2-mM TiCitrate, 250-mM KCl, and 100-µM D-methionine-methyl-d_3_ in 50-mM HEPES, pH 7.5. Assays were quenched by a twofold dilution in 100-mM sulfuric acid at each time point. 5′dAH, methionine, and *S*-adenosylhomocysteine were quantified after separation on an Agilent Technologies 1290 Infinity II series UHPLC system coupled to a 6470 QQQ Agilent Jet Stream electrospray-ionization mass spectrometer equipped with Agilent Zorbax Extend-C18 RRHD column (2.1 mm × 50 mm, 1.8-µm particle size). A standard curve of 5′dAH, Met, and SAH (500 nM through 300 µM) with 50-µM D-methionine-methyl-d_3_ (internal standard) was prepared for quantification using the Agilent MassHunter Quantitative Analysis 10.1 Software.

### Purification of MCR from *M. acetivorans* for crystallography

TAP-tagged MCR was purified under aerobic conditions as described (14) except that crystallography buffer composed of 100-mM Tris-HCl and 300-mM NaCl (pH 8) was used for washing steps after protein binding. Proteins were eluted using crystallography buffer containing 50mM biotin.

### Crystallization of *Ma*MCR Gln_420_ variant

The purified enzyme was concentrated to 25-mg/mL prior to crystallization via ultrafiltration. The crystallization trials utilized 2-µL sitting drops composed of 0.9:0.9:0.2 (protein:reservoir solution:additive screen) that were equilibrated against a 500-µL volume of the reservoir solution at room temperature. The reservoir solution contained 0.2-M ammonium acetate, 0.1-M sodium acetate (pH = 4), and 15%–20% (wt/vol) PEG 4000. Prior to freezing by vitrification in liquid nitrogen, crystals were soaked in reservoir solution supplemented with an additional 20% (vol/vol) glycerol or 30% (wt/vol) PEG 4000. Diffraction data were collected at MacCHESS using an Dectris Eiger2 detector. Raw diffraction images were integrated and scaled using either XDS or AutoProc. Molecular replacement was carried out using the coordinates for the structure of wild-type *Ma*MCR. The initial model was subject to manual rebuilding, and cofactors were added to the model after the free R factor dropped below 0.30. Relevant crystallographic statistics are detailed in Table S7.

### *Mm*MgmA structure determination by X-ray crystallography

RCN *Mm*MgmA crystals were generated via the hanging drop vapor diffusion method at room temperature by mixing 1 µL of a solution of *Mm*MgmA in storage buffer (5 mg/mL) with 1 µL of the well solution (0.2-M calcium chloride, 20% [wt/vol] PEG 3350, and 5-mM SAH). Crystals were prepared for data collection by mounting on rayon loops followed by soaking in cryoprotectant solution (50% [wt/vol] PEG 3350) and flash-freezing in LN. The structures were determined by single anomalous dispersion phasing using Autosol/HySS or by molecular replacement using the program PHASER (44). Model building and refinement were performed with Coot and phenix.refine, respectively (45, 46). Ligand geometric restraints were obtained from the Grade Web Server (https://www.globalphasing.com). Structures were validated and analyzed for Ramachandran outliers with the Molprobity server (47). Figures were prepared using PyMOL. Active site cavity mapping was prepared using Hollow (48).Data collection and refinement statistics are provided in Table S8.

## Data Availability

The structural data for *M. acetivorans* MCR containing the 2-methylglutamine modification and for *M. marburgensis* MgmA have been deposited in in Protein Data Bank (PDB) under the accession numbers 9ECN and 9CCB, respectively.

## SUPPLEMENTAL MATERIAL

Data set S1 mbio.03546-24-s0001.xlsx Amino acids found at McrA position 432 and coocurrence of MgmA.

Data set S2 mbio.03546-24-s0002.xlsx Presence of methylotropic genes and coocurrence of MgmA.

Supplemental Material mbio.03546-24-s0003.pdf Supplemental text, tables, and figures.

ASM does not own the copyrights to Supplemental Material that may be linked to, or accessed through, an article. The authors have granted ASM a non-exclusive, world-wide license to publish the Supplemental Material files. Please contact the corresponding author directly for reuse.
