# Supplementary material for: Genetic and biochemical characterization of a radical SAM enzyme required for post-translational glutamine methylation of methyl-coenzyme M reductase
Source: mBio. 2025 Jan 8;16(2):e03546-24. doi: 10.1128/mbio.03546-24 (PMC11796369; doi:10.1128/mbio.03546-24)
Supplement: Supplemental Material — Supplemental text, tables, and figures. [file mbio.03546-24-s0003.pdf]

Supplementary material

Genetic and biochemical characterization of a radical SAM enzyme required for post-translational glutamine methylation of methyl-coenzyme M reductase

Roy J Rodriguez Carrero,[a] Cody T. Lloyd,[b] Janhavi Borkar,[c] Shounak Nath,[d] Liviu M. Mirica,[d] Satish Nair,[c,e] Squire J. Booker,[b,f,g] William Metcalf[a,#]

[a]Department of Microbiology, University of Illinois at Urbana-Champaign

[b]Department of Chemistry, Pennsylvania State University

[c]Department of Biochemistry, University of Illinois, at Urbana-Champaign

[d]Department of Chemistry, University of Illinois, at Urbana-Champaign

[e]Center for Biophysics and Quantitative Biology, University of Illinois, at Urbana-Champaign

[f]Department of Biochemistry and Molecular Biology, Pennsylvania State University

[g]The Howard Hughes Medical Institute, Pennsylvania State University

[#]Address correspondence to: William W. Metcalf, metcalf@illinois.edu.

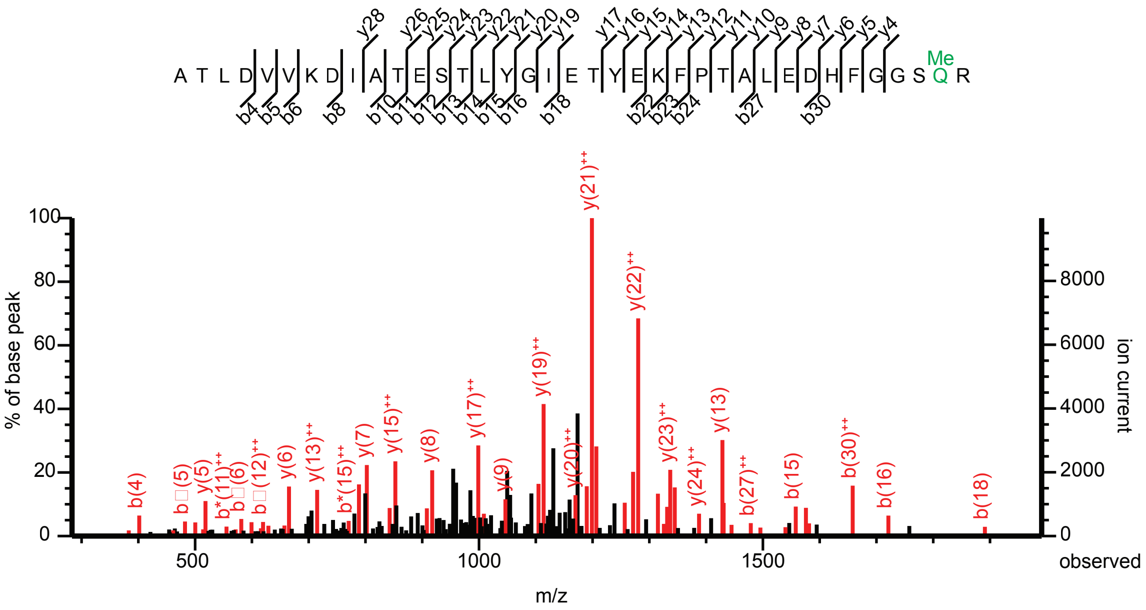

Figure S1. MS/MS analysis of the 1029 m/z tryptic peptide from the induced sample of *M. acetivorans* expressing Mh-MgmA. The sequence of the target peptide containing Gln_{420} (in green) is shown at the top, with the fragmentation sites for the "*b*" and "*y*" ions indicated. The selected ion chromatogram shown below depicts the m/z (Z = 4) and ion intensity of the "*b*" and "*y*" fragmentation ions that were automatically detected by MASCOT shown in red. The intact mass analysis of this peptide is shown in the top panel of Fig 2.

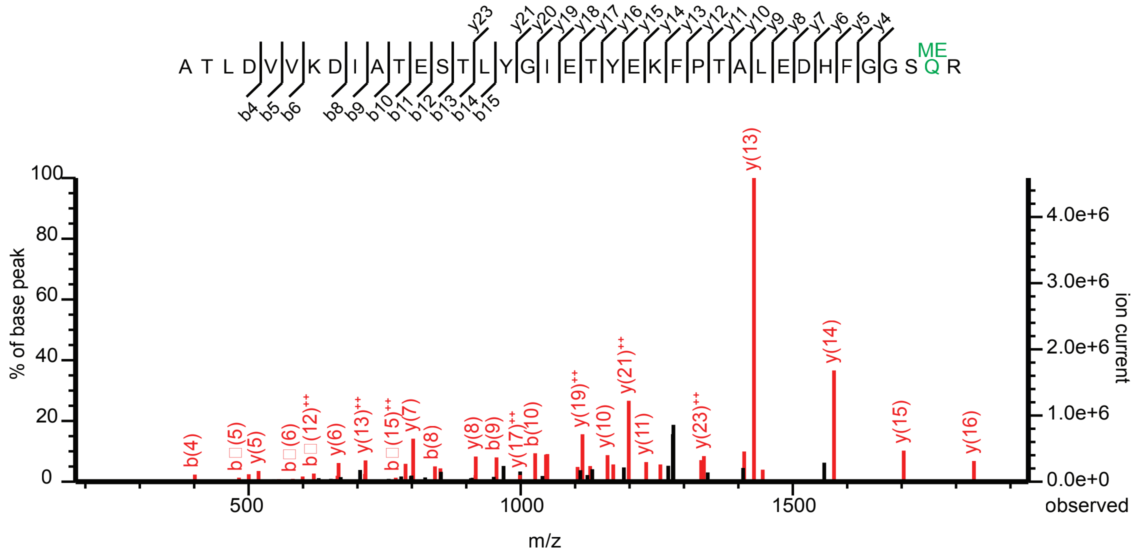

Figure S2. MS/MS analysis of the 1029 m/z tryptic peptide from the induced sample of McrA-edited *M. acetivorans* expressing Mh-MgmA. The sequence of the target peptide containing Gln_{420} (in green) is shown at the top, with the fragmentation sites for the "*b*" and "*y*" ions indicated. The selected ion chromatogram shown below depicts the m/z (Z = 4) and ion

intensity of the “*b*” and “*y*” fragmentation ions that were automatically detected by MASCOT shown in red. The intact mass analysis of this peptide is shown in the middle panel of Fig 2.

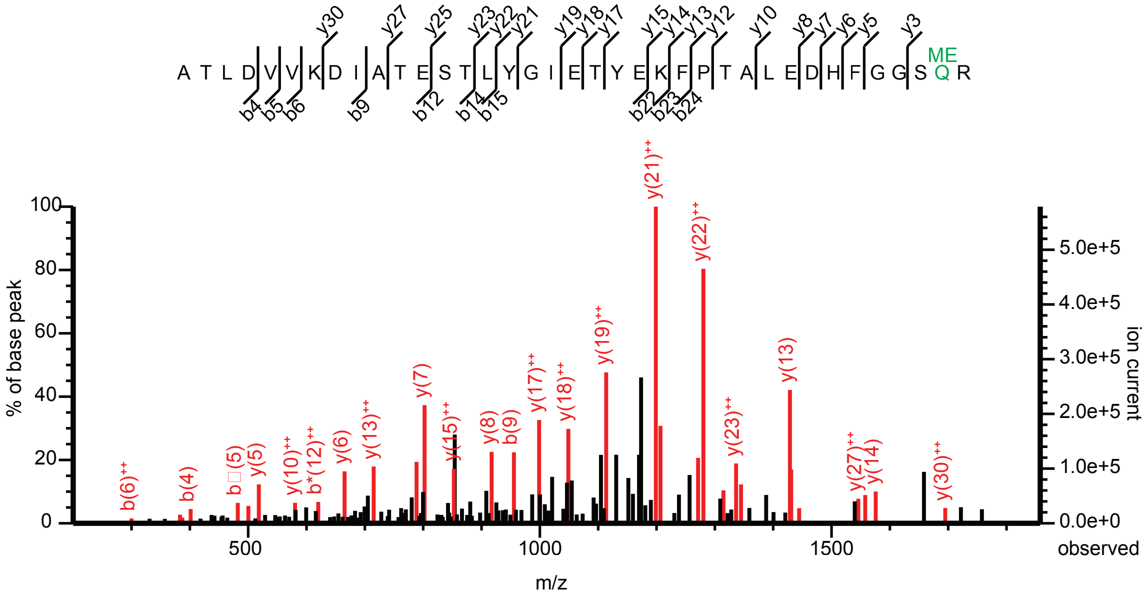

Figure S3. MS/MS analysis of the 1029 m/z tryptic peptide from the induced sample of *M. acetivorans* expressing Mm-MgmA. The sequence of the target peptide containing Gln_{420} (in green) is shown at the top, with the fragmentation sites for the “*b*” and “*y*” ions indicated. The selected ion chromatogram shown below depicts the m/z (Z = 4) and ion intensity of the “*b*” and “*y*” fragmentation ions that were automatically detected by MASCOT shown in red. The intact mass analysis of this peptide is shown in the bottom panel of Fig 2.

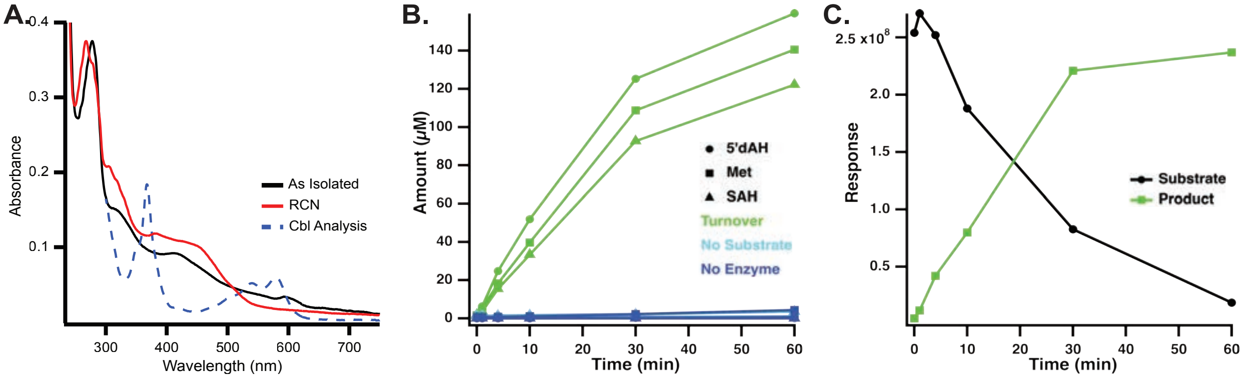

D. N E E Y P A L L L E D Q F G G S Q R A A V V A C

B & Y Series Name	Theoretical Mass	Observed Mass
B2	258.08518	258.0852001
B3	421.14851	421.1484001
B5	589.23838	589.2384001
Y6	585.359825	585.3599001
Y7	713.418405	713.4182001
Y8	800.450435	800.4505001
Y9	857.471895	857.4720001
Y10	914.493355	914.4936001
Y11	1061.561765	1061.561401
Y12	1189.620345	1189.621301
Y13	1304.647285	1304.648701
Y14	1433.689875	1433.690101
Y15	1546.773935	1546.775401
Y16	1659.857995	1659.858701
Y17	1730.895105	1730.896401
Y18	1827.947865	1827.949901

E. N E E Y P A L L L E D Q F G G S Q R A A V V A C

B & Y Series Name	Theoretical Mass	Observed Mass
B2	258.08518	258.0851001
B3	421.14851	421.1483001
B5	589.23838	589.2369001
B6	702.32244	702.3219001
B7	815.4065	815.4051001
Y6	585.359825	585.3596001
Y7	727.434055	727.4332001
Y8	814.466085	814.4656001
Y9	871.487545	871.4870001
Y10	928.509005	928.5089001
Y11	1075.577415	1075.577301
Y12	1203.635995	1203.636701
Y13	1318.662935	1318.663801
Y14	1447.705525	1447.705801
Y15	1560.789585	1560.790701
Y16	1673.873645	1673.874601
Y17	1744.910755	1744.913101
Y18	1841.963515	1841.964701

Figure S4. Biochemical characterization of *Mm*MgmA heterologously expressed and purified from *E. coli*. Panel A: *Mm*MgmA UV-Vis characterization of the protein after initial purification (as isolated, black trace), chemical reconstitution of the cofactors (RCN, red trace), and analysis of Cbl extracted from the RCN protein (dashed blue trace). Panel B: Time-dependent formation of 5′dAH (circle), methionine (Met, square), and S-adenosylhomocysteine (SAH) during activity assays in the absence of enzyme (dark blue trace), absence of peptide substrate (light blue trace), and under turnover conditions (green trace). Panel C: Time-dependent decay of the substrate peptide (black trace) and formation of the methylated product peptide (green trace) during activity assays under turnover conditions (Panel B, green traces) monitored by high-resolution MS. MS/MS analysis of the substrate peptide (Panel D.) and product peptide (Panel E.) to observe the “B” and “Y” ion fragmentation series. Observed masses reveal that MmMgmA appends a methyl group (Δm/z = 14.0150) to the Gln420 (Panel E, Y7 ion).

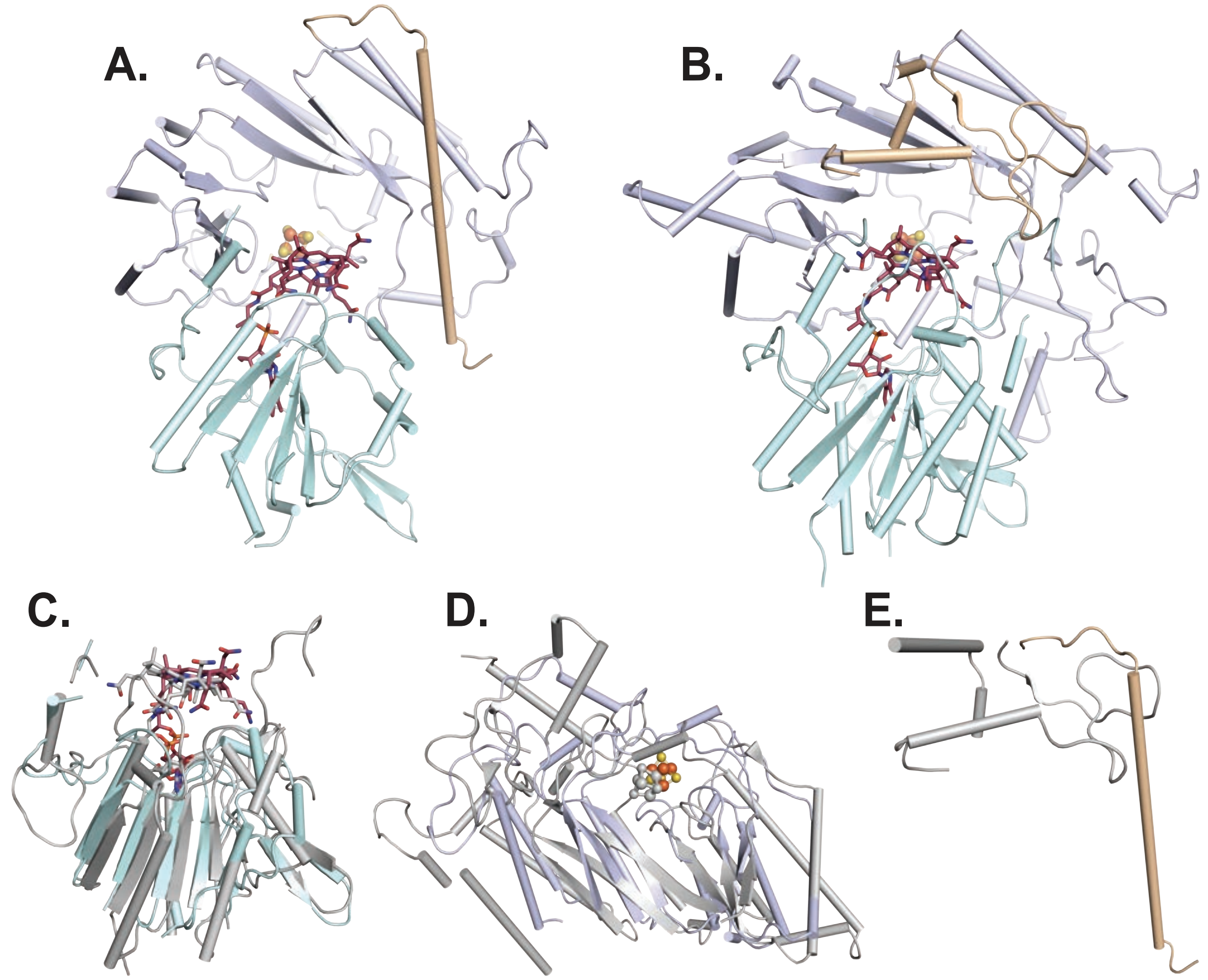

Figure S5. Structural comparison of *Mm*MgmA to the Class B rSAM methyltransferases TsrM. Ribbon diagram of *Mm*MgmA (Panel A) and TsrM (Panel B, PDB 6WTE) with domains colored to highlight structural similarities: Rossman fold (pale cyan), ¾ TIM Barrel (light blue), and C-terminal domain (wheat).(REF) Panel C. Overlays of the Rossman fold (Panel C), ¾ TIM Barrel (Panel D), and the C-terminal domain (Panel E) of MmMgmA (pale cyan, light blue, and wheat respectively) with TsrM in (grey).

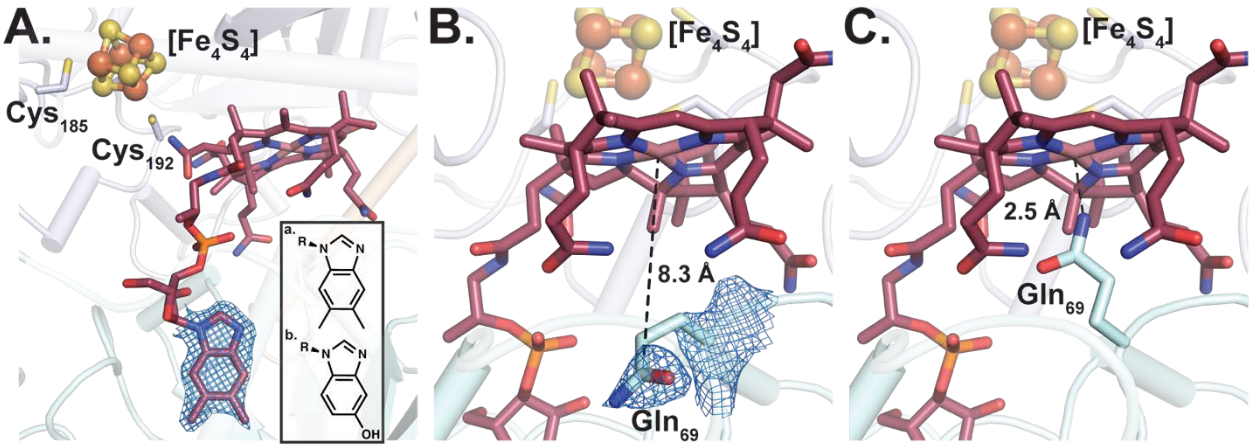

Figure S6. *Mm*MgmA functionally binds the dimethylbenzimidizole-containing cobalamin. Panel A: Close-up of the rSAM [Fe_4S_4] cluster and cobalamin cofactor bound in the active site of *Mm*MgmA. The electron density map (2Fo-Fc, contoured at 1.5σ) reveals that *Mm*MgmA can bind the non-native dimethylbenzimidizole-containing Cbl (insert a) when heterologously expressed and purified from *E. coli* as opposed to the native hydroxybenzimidizole-containing cobamide (insert b.) found in methanogens. Panel B: Analysis of the proximal face reveals that Cbl does not have a lower axial ligand. The closest residue to the cobalt, Gln_{69}, is 8.3 Å away. Panel C. Rotation around the β-carbon of Gln_{69} places the amine within coordinating distance to Cbl (2.5 Å).

```
1HBM_A         -ADKLFINALKKKFEESPEEK-----------------KTTFYTLGGWKQSERKTEFVNA   42
WP_013296337 MADKLFINALKKKFEESPEEK-----------------KTTFYTLGGWKQSERKTEFVNA   43
Mac McrA       MAADIF-----AKFKKSMEVKFTQEYGSNKQAGGDITGKTEKFLRLGPEQDARKQEMIKA   55

1HBM_A         GKEVAAKRGIPQYNP--DIGTPLGQRVLMPYQVSTTDTYVEGDDLHFVNNAAMQQMWDDI  100
WP_013296337 GKEVAAKRGIPQYNP--DIGTPLGQRVLMPYQVSTTDTYVEGDDLHFVNNAAMQQMWDDI  101
Mac McrA       GKEIAEKRGIAFYNPMMHMGAPLGQRAITPYTISGTDIVAEPDDLHYVNNAAMQQMWDDI  115

1HBM_A         RRTVIVGLNHAHAVIEKRLGKEVTPETITHYLETVNHAMPGAAVVQEHMVETHPALVADS  160
WP_013296337 RRTVIVGLNHAHAVIEKRLGKEVTPETITHYLETVNHAMPGAAVVQEHMVETHPALVADS  161
Mac McrA       RRTCIVGLDMAHETLEKRLGKEVTPETINHYLETLNHAMPGAAVVQEMMVETHPALVDDC  175

1HBM_A         YVKVFTGNDEIADEIDPAFVIDINKQFPEDQAETLKAEVGDGIWQVVRIPTIVSRTCDGA  220
WP_013296337 YVKVFTGNDEIADEIDPAFVIDINKQFPEDQAETLKAEVGDGIWQVVRIPTIVSRTCDGA  221
Mac McrA       YVKIFTGDDELADEIDKQYVINVNKMFSEEQAAQIKASIGKTTWQAIHIPTIVSRTTDGA  235

1HBM_A         TTSRWSAMQIGMSMISAYKQAAGEAATGDFAYAAKXAEVIHMGTYLPVRXARGENEPGGV  280
WP_013296337 TTSRWSAMQIGMSMISAYKQAAGEAATGDFAYAAKHAEVIHMGTYLPVRRARGENEPGGV  281
Mac McrA       QTSRWAAMQIGMSFISAYAMCAGEAAVADLSFAAKHAALVSMGEMLPARRARGPNEPGGL  295

1HBM_A         PFGYLADICQSSRVNYEDPVRVSLDVVATGAMLYDQIWLGSYMSGGVGFTQYATAAYTDN  340
WP_013296337 PFGYLADICQSSRVNYEDPVRVSLDVVATGAMLYDQIWLGSYMSGGVGFTQYATAAYTDN  341
Mac McrA       SFGHLSDIVQTSRVS-KDPAKIALEVVGAGCMLYDQIWLGSYMSGGVGFTQYATAAYTDD  354

1HBM_A         ILDDFTYFGKEYVEDKYGLC-------EAPNNMDTVLDVATEVTFYGLEQYEEYPALLED  393
WP_013296337 ILDDFTYFGKEYVEDKYGLC-------EAPNNMDTVLDVATEVTFYGLEQYEEYPALLED  394
Mac McrA       ILDNNTYYDVDYINDKYNGAANLGTDNKVKATLDVVKDIATESTLYGIETYEKFPTALED  414

1HBM_A         QFGGSXRAAVVAAAAGCSTAFATGNAQTGLSGWYLSMYLHKEQHSRLGFYXYDLQDQXGA  453
WP_013296337 QFGGSQRAAVVAAAAGCSTAFATGNAQTGLSGWYLSMYLHKEQHSRLGFYGYDLQDQCGA  454
Mac McrA       HFGGSQRATVLAAASGVACALATGNANAGLSGWYLSMYVHKEAWGRLGFFGFDLQDQCGA  474

1HBM_A         SNVFSIRGDEGLPLELRGPNYPNYAMNVGHQGEYAGISQAPHAARGDAFVFNPLVKIAFA  513
WP_013296337 SNVFSIRGDEGLPLELRGPNYPNYAMNVGHQGEYAGISQAPHAARGDAFVFNPLVKIAFA  514
Mac McrA       TNVLSYQGDEGLPDELRGPNYPNYAMNVGHQGGYAGIAQAAHSGRGDAFTVNPLLKVCFA  534

1HBM_A         DDNLVFDFTNVRGEFAKGALREFEPAGERALITPAK                         549
WP_013296337 DDNLVFDFTNVRGEFAKGALREFEPAGERALITPAK                         550
Mac McrA       DELMPFNFAEPRREFGRGAIREFMPAGERSLVIPAK                         570
```

Figure S7. Alignment of McrA sequences from *M. acetivorans* and *M. marburgensis*. Due to differences in the numbering systems used in the protein sequences found in various publications, it can be difficult to directly compare analogous residues. The alignment shows the numbering system of *M. acetivorans* McrA (Mac McrA) that is used in Figure 5, along with the numbering of *M. marburgensis* McrA (WP_013296337), as well as the numbering system of McrA from the *M. marburgensis* crystal structure (1HMB A). The position of residues shown in Figure 5 are color coded as follows: Gln_{161}, pink; Tyr_{346}, green; (α-methyl)-Gln_{420}, red; Thr_{423}/Ala_{403}, cyan.

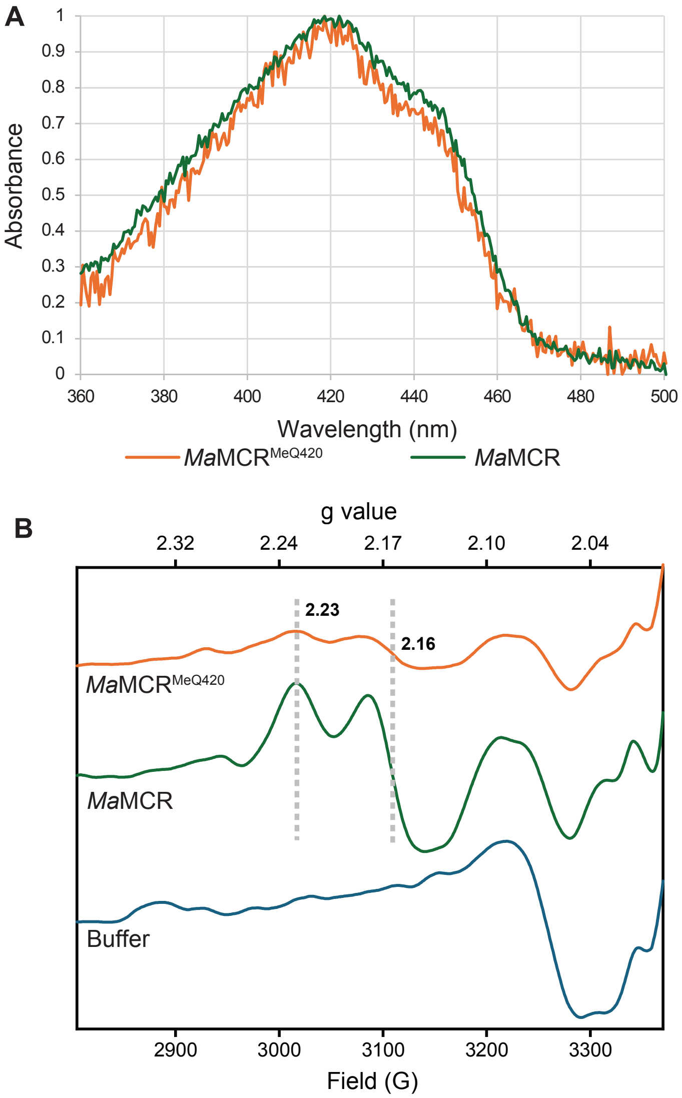

Figure S8. UV-VIS and EPR spectroscopy of MCR from *M. acetivorans* expressing *Mm*MgmA. Panel A: The UV-VIS spectrum of Gln_{420}-methylated MCR from *M. acetivorans* purified under reducing conditions (*Ma*MCR^{MeQ420}) compared to the unaltered MCR purified under the same conditions (*Ma*MCR). Both spectra contain the peak a 420nm and shoulder at 440nm typically associated with the Ni(II) MCRsilent variant. The absorbance for both variants has been normalized on a 0 to 1 scale. Panel B: The EPR spectrum of MaMCR^{MeQ420} and *Ma*MCR purified under reducing conditions. Samples were prepared in 100 mM TrisHCl buffer at pH 8 with 50% glycerol containing 10 mM DTT, 300 mM NaCl, 100 uM Ti citrate and 50 mM biotin. Both spectra exhibit g anisotropies at gx,y = 2.16 and gz = 2.23 typically associated to that of an MCRox1 species, which is indicative of similar electronic structure at the Ni center in both forms. Spin integration with respect to a Cu(II) standard revealed an odd oxidation state Ni concentration of 2.6 uM and 3.8 uM for the MaMCRMeQ420 and MaMCR, which equates to 50% and 26%, respectively, of each proetin.The EPR spectra have been zoomed in to separate the strong Ti(III) EPR signal at g ≈ 1.97 from the Ni EPR signals. EPR experimental conditions: frequency = 9.420 GHz, power = 0.63 mW, modulation frequency = 100 kHz, modulation amplitude = 5 G, time constant = 0.082 s.

Table S1. Gene families present in *Methanothermococcus marburgensis*, *Methanocaldococcus jannaschii*, *Methanotorris formicicus* and *Methanopyrus kandleri* that are absent in *Methanosarcina acetivorans* and *Methanosarcina barkeri*.[a]

	Locus Tag (*M. marburgensis*)	Locus Tag (*M. jannaschii*)	Locus tag (*M. formicicus*)	Locus Tag (*M. kandleri*)	Gene Annotation
1	MTBMA_c00650	MJ0212	Metfo_0533	MK0715	nucleoid protein Alba
2	MTBMA_c00880	MJ0033	Metfo_0364	MK0828	thiol-driven fumarate reductase, flavoprotein subunit (EC 1.3.99.-)
3	MTBMA_c00960	MJ1142	Metfo_0913	MK1680	arsenite efflux ATP-binding protein ArsA (TC 3.A.4.1.1)
4	MTBMA_c01230	MJ0832	Metfo_1959	MK0901	ribonucleoside-triphosphate reductase class III catalytic subunit (EC 1.17.4.2)
5	MTBMA_c01770	MJ1612	Metfo_1358	MK1193	phosphoglycerate mutase (EC 5.4.2.1)
6	MTBMA_c01870	MJ1634	Metfo_2013	MK1076	hypothetical protein
7	MTBMA_c02190	MJ0104	Metfo_1462	MK0070	DNA helicase
8	MTBMA_c02240	MJ0612	Metfo_1807	MK0798	prephenate dehydrogenase
9	MTBMA_c02450	MJ1351	Metfo_1497	MK0550	glutamate synthase, alpha subunit related protein
10	MTBMA_c02500	MJ0119	Metfo_0406	MK0811	hypothetical protein
11	MTBMA_c02650	MJ0299	Metfo_1378	MK0954	fructose 1,6-bisphosphatase
12	MTBMA_c02810	MJ0781	Metfo_0711	MK0707	ATPase
13	MTBMA_c03110	MJ0264	Metfo_0076	MK0084	HycB-related protein
14	MTBMA_c03730	MJ0294	Metfo_1030	MK1035	ATP-dependent helicase
15	MTBMA_c04210	MJ0092	Metfo_0474	MK0132	thiol:fumarate reductase, subunit B
16	MTBMA_c04540	MJ1482	Metfo_2008	MK0868	2-phosphoglycerate kinase (EC 2.7.1.-)
17	MTBMA_c04550	MJ0922	Metfo_1260	MK0869	hypothetical protein
18	MTBMA_c04660	MJ0261	Metfo_2020	MK1503	hypothetical protein
19	MTBMA_c05040	MJ0468	Metfo_0202	MK1220	30S ribosomal protein S4e
20	MTBMA_c05060	MJ0469.1	Metfo_0200	MK1222	SSU ribosomal protein S14P
21	MTBMA_c05160	MJ0479	Metfo_0190	MK0025	adenylate kinase
22	MTBMA_c05180	MJ0655	Metfo_0463	MK0023	LSU ribosomal protein L34E
23	MTBMA_c05200	MJ0657	Metfo_0465	MK0833	LSU ribosomal protein L14E
24	MTBMA_c05920	MJ1616	Metfo_0659	MK1600	inosine-5'-monophosphate dehydrogenase (EC 1.1.1.205)
25	MTBMA_c06400	MJ0490	Metfo_0512	MK1069	malate dehydrogenase
26	MTBMA_c06570	MJ0885	Metfo_1369	MK1039	replicative DNA polymerase I (EC 2.7.7.7)
27	MTBMA_c06580	MJ1437	Metfo_0607	MK0970	hydrolase
28	MTBMA_c06980	MJ1501	Metfo_1010	MK0233	reduced coenzyme F420:NADP oxidoreductase (EC 1.6.99.-)
29	MTBMA_c07290	MJ0870	Metfo_0645	MK0799	F420-dependent sulfite reductase

30	MTBMA_c07850	MJ0527	Metfo_1190	MK0476	membrane-bound hydrogenase subunit ehaB
31	MTBMA_c07890	MJ0524	Metfo_1186	MK0472	membrane-bound hydrogenase subunit ehaF
32	MTBMA_c07900	MJ0523	Metfo_1185	MK0471	membrane-bound hydrogenase subunit ehaG
33	MTBMA_c07910	MJ0522	Metfo_1184	MK0470	membrane-bound hydrogenase subunit ehaH
34	MTBMA_c07930	MJ0520	Metfo_1182	MK0468	membrane-bound hydrogenase subunit ehaJ
35	MTBMA_c07950	MJ0518	Metfo_1180	MK0466	membrane-bound hydrogenase subunit ehaL
36	MTBMA_c07980	MJ0515	Metfo_1177	MK0463	membrane-bound hydrogenase subunit ehaO
37	MTBMA_c08290	MJ1399	Metfo_1771	MK0400	dephospho-CoA kinase
38	MTBMA_c08510	MJ0542	Metfo_0959	MK0252	phosphoenolpyruvate synthase
39	MTBMA_c09430	MJ0708	Metfo_1755	MK0638	pre-rRNA-processing protein TSR3
40	MTBMA_c09510	MJ1246	Metfo_2026	MK0732	succinyl-CoA synthase, subunit alpha
41	MTBMA_c09520	MJ1676	Metfo_1946	MK0634	hypothetical protein
42	MTBMA_c10800	MJ0827	Metfo_1139	MK0847	hypothetical protein
43	MTBMA_c11230	MJ1526	Metfo_1867	MK0206	hypothetical protein
44	MTBMA_c11260	MJ0949	Metfo_1292	MK0454	hypothetical protein
45	MTBMA_c11460	MJ1374	Metfo_0246	MK0284	metal-dependent hydrolase
46	MTBMA_c11710	MJ1473	Metfo_0008	MK0667	methionine synthase (B12-independent) (EC 2.1.1.14)
47	MTBMA_c12620	MJ1480	Metfo_0034	MK0866	lysine-oxoglutarate reductase/saccharopine dehydrogenase
48	MTBMA_c12840	MJ0319	Metfo_1383	MK1384	hypothetical protein
49	MTBMA_c13450	MJ0221	Metfo_0098	MK1013	A1AO ATPase, subunit K
50	MTBMA_c14080	MJ0817	Metfo_1578	MK0162	phosphatidylserine decarboxylase
51	MTBMA_c14130	MJ0408	Metfo_2087	MK0786	hypothetical protein
52	MTBMA_c14160	MJ0537	Metfo_0032	MK0729	2-oxoglutarate ferredoxin oxidoreductase, beta subunit (EC 1.2.7.3)
53	MTBMA_c14170	MJ0536	Metfo_0033	MK0730	2-oxoglutarate ferredoxin oxidoreductase, gamma subunit (EC 1.2.7.3)
54	MTBMA_c14180	MJ0210	Metfo_0251	MK0731	succinyl-CoA synthetase (ADP-forming) beta subunit (EC 6.2.1.5)
55	MTBMA_c14940	MJ1244	Metfo_1975	MK1106	transcriptional regulatory protein
56	MTBMA_c15050	MJ1077	Metfo_1903	MK1460	seryl-tRNA synthetase (EC 6.1.1.11)
57	MTBMA_c15140	MJ0118	Metfo_1524	MK0652	methyl-coenzyme M reductase II, subunit delta
58	MTBMA_c15180	MJ1191	Metfo_1553	MK0267	F420-non-reducing hydrogenase, subunit G
59	MTBMA_c15200	MJ0787	Metfo_0700	MK0017	Hmd co-occurring protein HcgG

60	MTBMA_c15220	MJ0005	Metfo_0765	MK0322	formate dehydrogenase, beta subunit (F420) (EC 1.2.99.-)
61	MTBMA_c15260	MJ0784	Metfo_0707	MK0013	H2-forming methylenetetrahydromethanopterin dehydrogenase (EC 1.12.98.2)
62	MTBMA_c15270	MJ0785	Metfo_0706	MK0016	Hmd co-occurring protein HcgA
63	MTBMA_c15280	MJ0488	Metfo_0069	MK0018	Hmd co-occurring protein HcgB
64	MTBMA_c15310	MJ0693	Metfo_1725	MK0010	Hmd co-occurring protein HcgE
65	MTBMA_c15540	MJ0865	Metfo_0529	MK0797	hypothetical protein
66	MTBMA_c15830	MJ1425	Metfo_1323	MK0392	malate dehydrogenase (NAD) (EC 1.1.1.37)/L-sulfolactate dehydrogenase (EC 1.1.1.272)
67	MTBMA_c15840	MJ0255a	Metfo_0143	MK0395	sulfopyruvate decarboxylase subunit alpha (EC 4.1.1.79)
68	MTBMA_c16230	MJ1458	Metfo_1414	MK0609	membrane-bound hydrogenase subunit ehbQ
69	MTBMA_c16280	MJ1302	Metfo_1465	MK0462	membrane-bound hydrogenase subunit ehbL
70	MTBMA_c16520	MJ1105	Metfo_0847	MK1011	potassium uptake protein TrkA
71	MTBMA_c16670	MJ0188	Metfo_1296	MK0907	CBS domain containing protein
72	MTBMA_c16860	MJ0029	Metfo_1555	MK0931	coenzyme F420-reducing hydrogenase, alpha subunit (EC 1.12.98.1)
73	MTBMA_c17160	MJ1119	Metfo_0218	MK0434	diaminopimelate epimerase (EC 5.1.1.7)
74	MTBMA_c17440	MJ0649	Metfo_1245	MK0881	dehydrogenase
75	MTBMA_c17670	MJ1597	Metfo_1603	MK0122	serine hydroxymethyltransferase (EC 2.1.2.1)
76	MTBMA_c17770	MJ0921	Metfo_1537	MK0351	tRNA(Ile2) 2-agmatinylcytidine synthetase
77	MTBMA_c17840	MJ1552	Metfo_0044	MK1377	nucleic acid-binding protein

Table S2. List of plasmids used in this study

Plasmid	Features	Source
**pAMG40**	Vector for fosmid retrofitting that contains pC2A and λattB	(1)
**pJK031A**	Vector with P*mcrB*(*tetO1*) promoter fusion to *uidA* that contains φC31-attP and λattP	(1)
**pDN201**	pJK027A-derived plasmid with P*mcrB*(*tetO1*) promoter fusion to Spy *cas9*	(2)
**pRR007**	pDN201-derived plasmid with a synthetic fragment containing P*mtaCB1* promoter fusion to an sgRNA targeting the MA4546(*mcrA*) locus in *M. acetivorans*	This study
**pRR008**	pRR007-derived plasmid with a homology template comprising of 1 kb upstream and downstream flanks to generate a T to A amino acid substitution in the MA4546 (*mcrA)* locus in *M. acetivorans*	This study
**pRR009**	pRR007-derived plasmid with a homology template comprising of 1 kb upstream and downstream flanks to generate A to S, T to A and L to M amino acid substitutions in the MA4546 (*mcrA)* locus in *M. acetivorans*	This study
**pRR011**	Cointegrate of pRR008 and pAMG40	This study
**pRR012**	Cointegrate of pRR009 and pAMG40	This study
**pRR013**	pJK031A-derived plasmid with P*mcrB*(*tetO1*) promoter fusion to Mh-*mgmA*	This study
**pRR026**	pJK031A-derived plasmid with P*mcrB*(*tetO1*) promoter fusion to Mm-*mgmA*	This study
**pRR029**	pJK031A-derived plasmid with with P*mcrB*(*tetO1*) promoter fusion to Mm-*mgmA* with a TEV cleavable tandem affinity purification (TAP) tag comprising of a 3X FLAG tag and a Twin-Strep tag on its N-terminus	This study
**pRR032**	Cointegrate of pRR026 and pAMG40	This study
**pRR035**	Cointegrate of pRR029 and pAMG40	This study
**pRR037**	pJK031A-derived plasmid with P*mcrB*(*tetO1*) promoter fusion to inactive Mm-*mgmA*	This study

Table S3. List of primers used in this study.

Primer	Sequence (bold underlined sequence represents overhangs for HiFi assembly)
For making PCR amplicon of Mm-MgmA for Gibson Assembly	

**Mmar_MgmA_F**	**TGATTTTAATAAATTAAGGAGGAAATTCAT**ATGACTCGTGTGGTTGTTTT
**Mmar_MgmA_R**	**CATACATTATACGAAGTTATCAAGAAGCTT**CTAAAGTTCCCTCTTCAACA
For making PCR amplicon of inactive Mm-MgmA for Gibson Assembly	
**MgmA_Alasub_F**	**TCGTGGTGCTCCGGGTAATGCTACCTTTGC**TCAGGTTCCGGAATTTTTTGG
**MgmA_Alasub_R**	**GCAAAGGTAGCATTACCCGGAGCACCACGA**TGGGTTTCAATATACACGCT
For making PCR amplicon of 3X FLAG tag and a Twin-Strep tag for Gibson Assembly	
**F_Ntag_MgmA_1**	**TGATTTTAATAAATTAAGGAGGAAATTCAT**ATGGACTATAAGGACCACGAC
**MgmA_Mmar_Nter_Strep_R**	**TTCCGGTGTCAGAACAACAACACGGGTCAT**ACCTTGAAAATAGAGATT

Table S4. List of synthetic DNAs used in this study.

***M. harundinacea* MgmA gBlock**

TGATTTTAATAAATTAAGGAGGAAATTCATATGAAGACGACGATCGTATCCCCTAAGATCTACA
CCTACGGCTCCCTAGTCCTCGGCGGCATCCTCAGGGACCGGGGCCACGCCGTCTCGATCA
CCAGGGACCTCTCCTCCGAGGGGGATCTGACCCTCCTCAGCCTCTTCTCCACCTCCCAGC
TCCTCGACCCCGAGATCCGGGAGTTGGCGAGCCGGGCTCCGAGGATATACGTCGGAGGC
CCCGTCGGCCTCGTCCCCGAGATCGTCCTCGGGGAGCTGGAGGTAGATGCTGTGGTCGT
GGGGGAGGGGGAGGAGGTCGTCGCAGACCTAGTCGAAGGGGGGCCCTCCGAAGAGATC
TCCGGCCTAGCCTTCCTCCGGGACGGCGTGGTGGTCAAGACCGATCCCATCCCCGTCTCC
GACCTCGACCACGTCATGCCCCTCATCCCCGACGACCTGAGGAGCCAGAGCGTCCGGGG
AGCCAACGTCTACATCGAGACCCACCGGGGATGCCTGGGAGGCTGCACCTTCTGCCAGGT
CCCCCGGTTCTTCGGCCGGTCAATCCGGTCCCGGTCCCTCGAGAACATCCTCGCCGAGGT
CAGAGAGATGAAGAGGAGGGGGGTCACCAGGGTGGCGGTCAGCGGCGGTACGGGATCAC
TATTCGGCTACGGGAAGGAGGTGAACAAGGAGGCTTTCATCACCCTCTTAAAGGGCCTGGC
TGAGATCCTGGGTCCAAAGAACCTCTCAGTCCCGGATATGAGGGTCGACTTCGTCGACGAG
GAGATCCTGGAGGCGGTGAGGAGGTACACCGTCGGCTGGGTCTTCTTCGGGATCGAGTC
GGGGAGCCCCGGGATCCTCAGGGCGATGAAGAAGGGGACGACCCCCGAGAAGAGCCTG
GAGGCGGTGGAGCTCGCCCACCAGTCCGGGGTCAAGGTCGGCGGAAGCTTCATCGTCGG
CTACCCCGGCGAGATGGAGGAGGACTACCAGGCGACCCTAGACTTCATGGAGGAGGCGAT
GCTGGAGGACGTCTTCGCGAGCGTCGCAGAGCCGATACCAGGAACTCCCCTCGCCAAGAT
CGCCCTGGACCTGCCCCGGGAGGATAACCCCCTCTACCGGGAGCATGCCGGAGAATATCG
GGCCCTGAGGATCTCGGAGGCGGAGGCCCGGTGCTTCAACCTGATGCTCACCGGGATGA
GCTGCAAGCCGGTGCCGCGAATAGTCGACGACGCCACCTACTCCGCCTTCCTTCAGGAGG

TGCGAACTCAGGGGAGGGATGTGAGGAGGGTGATGGGCCTTCTGGAGAAATATAAGGACC
GGATCGTCTGAAAGCTTCTTGATAACTTCGTATAATGTATG

McrA edit gBlock

TTTTTTTCGAAGTTTAAACCTGCAGGCGCGAACAACATCAGTCACCTAAAAAGAGAAAACGA
ATTACACGATCACTAATTTTAAATTTTATATATGTTGACTGAGATTGCAAATTTGAACATTGAAA
TTTTTTACCCGCTTGATCTGAATAATGACATTGTTCAAAAAAAGTACAAATGATAAAAAAGAAA
GCTTCTCAAAAAACAGTAAAGAAGTTCTCCCCAAAATCACCTCAAAAATTCAGAGCTCTATTA
TCAGAAAAAGCGAGCTTAAAAAATTCAAAGGAAAGATACCCCTCTGCACCCTCAAAATTTTA
GACCCTGTGTTGACCTGTAAAAATCAGGAAAAAATTTCCGTCGGTTATGGTATATGTGATGAT
TTCCCCTAATTATGCTGTAAGCACATGTACCGGATGCAGCTGCGAGCAGTTTTAGAGCTAGA
AATAGCAAGTTAAAATAAGGCTAGTCCGTTATCAACTTGAAAAAGTGGCACCGAGTCGGTGC
TTTTGCCCTCAGTTCTCTTTTTCTTTTTCTTAAACTTCACGCACTGCACTTTTGTCCTCACTTT
TTTCATGCCGTCAGATTAACTACTTTTTCTATCCTTGAAATCAGCGGCTTTTCAGCCCTCATG
TAGGCGCGCCGGCGATCGCGGCCGCTTAATTAAT

HDR *M. harundinacea* change gBlock

TCCTTTTGGAGCCTTTTTTTTTCGAAGTTTTGTCCTTCGCTGCAAAGCACGCAGCCCTTGTC
TCCATGGGTGAAATGCTCCCCGCAAGGCGTGCCCGCGGACCAAACGAGCCCGGTGGACT
TTCCTTCGGTCACCTCTCAGACATCGTCCAGACAAGCCGTGTATCCAAAGACCCCGCAAAG
ATTGCCCTTGAAGTAGTCGGCGCAGGCTGTATGCTCTACGACCAGATCTGGCTCGGATCCT
ACATGTCCGGTGGTGTCGGGTTCACCCAGTATGCAACTGCTGCATACACCGATGACATCCT
CGACAACAACACCTACTATGACGTTGACTACATCAACGACAAGTACAACGGTGCTGCAAATC
TGGGCACTGACAACAAGGTTAAGGCAACCCTCGACGTCGTAAAGGACATCGCAACCGAGT
CCACACTCTACGGTATCGAGACCTACGAGAAATTCCCGACTGCCCTTGAAGACCACTTCGG
TGGATCCCAGAGATCCGCGGTCATGGCCGCCGCCTCCGGTGTTGCATGTGCCCTTGCAAC
CGGAAACGCCAACGCTGGTCTCTCCGGCTGGTACCTCTCCATGTATGTCCACAAGGAAGCA
TGGGGCCGCCTCGGCTTCTTCGGTTTCGACCTGCAGGACCAGTGTGGTGCCACAAACGTT
CTGTCCTACCAGGGCGACGAAGGTCTCCCAGACGAACTCCGTGGTCCAAACTACCCGAAC
TACGCAATGAACGTCGGTCACCAGGGCGGATACGCAGGTATCGCTCAGGCAGCCCACTCA
GGCCGCGGCGACGCATTCACCGTCAACCCGCTCCTCAAGGTCTGCTTCGCTGACGAACTC
ATGCCCTTCAACTTCGCAGAGCCAAGGAGAGAGTTCGGCCGCGGTGCCATCAGAGAGTTC
ATGCCTGCTGGTGAGAGATCCCTCGTCATCCCGGCAAAATAAACTCAATAAATCAAACACTT
AAACCTGCAGGCGCGAACAACATCAGTCAC

Table S5. List of target sequences used in this study

Locus tag	Target sequence (bold underlined sequence represents the PAM)
MA4546 (*mcrA*)	ACCGGATGCAGCTGCGAGCA**CGG**

Table S6. List of *Methanosarcina acetivorans* strains used in this study.

Strain	Genotype	Construction details	Source
**WWM60**	Δ*hpt*::*PmcrB-tetR*	---	(1)
**WWM75**	Δ*hpt*::*PmcrB-tetR-ΦC31-int-attB*	---	(1)
**WWM1086**	Δhpt::PmcrB-tetR, Enterokinase cleavable TAP-tag at N-terminus of mcrG	---	(3)
**WWM1145**	Δ*hpt*::*PmcrB-tetR-ΦC31-int-attB::pRR013*	WWM75 was transformed to Pur^R with pRR013	This study
**WWM1169**	Δ*hpt*::*PmcrB-tetR- ΦC31-int-attB*<br>*mcrA* (A1267G C1269A)	Cas9-dependent gene editing of *mcrA* in WWM75 using pRR011 to match M. concilii local (AA residues)	This study
**WWM1170**	Δ*hpt*::*PmcrB-tetR- ΦC31-int-attB*<br>*mcrA* (G1264T, A1266C, A1267G, C1269G, G1272C, C1273A, C1275G, A1278C, T1281C, A1282C)	Cas9-dependent gene editing of *mcrA* in WWM75 using pRR011 to match M. harundinacea local (AA residues)	This study
**WWM1177**	Δ*hpt*::*PmcrB-tetR- ΦC31-int-attB::pRR013*<br>*mcrA* (A1267G, C1269A)	WWM1169 was transformed to Pur^R with pRR013	This study
**WWM1178**	Δ*hpt*::*PmcrB-tetR- ΦC31-int-attB::pRR13*<br>*mcrA* (G1264T, A1266C, A1267G, C1269G, G1272C, C1273A, C1275G, A1278C, T1281C, A1282C)	WWM1170 was transformed to Pur^R with pRR013	This study
**WWM1184**	Δ*hpt*::*PmcrB-tetR-ΦC31-int-attB::pRR026*	WWM75 was transformed to Pur^R with pRR026	This study

**WWM1185**	Δ*hpt*::*PmcrB-tetR/pRR035*	WWM60 was transformed to Pur^R with pRR035	This study
**WWM1186**	Δhpt::PmcrB-tetR, Enterokinase cleavable TAP-tag at N-terminus of mcrG/pRR032	WWM1086 was transformed to PurR with pRR032	This study
**WWM1187**	Δ*hpt*::*PmcrB-tetR-ΦC31-int-attB::pRR037*	WWM75 was transformed to Pur^R with pRR037	This study

Table S7. Data Collection and Refinement Statistics for *Ma*MCR$^{me\text{-}Gly420}$

	***Ma*MCR$^{me\text{-}Gly420}$**
**Data collection**	
Wavelength (Å)	0.9686
Space group	$P2_1$
Unit Cell (Å/degrees)	109.5, 82.9, 122.5/ 93.8
Resolution range (Å) [1]	50.-2.0 (2.1-2.0)
Total reflections	696,802
Unique reflections	147,424
Multiplicity	4.7 (4.9)
Completeness (%)	99.5 (95.6)
Mean I/sigma (I)	8.9 (2.9)
R-merge (%)[2]	12.2 (60.2)
R-pim (%)	9.8 (49.0)
CC ½	0.994 (0.781)
**Refinement**	
Resolution (Å)	25.0-2.0
Number of reflections	140,019
R-work	15.2
R-free[3]	19.3
Number of atoms	
Macromolecules	18,750
Co-factors	284
Solvent	1,385
Average B-factor	
Macromolecules	26.4
Co-factors	24.0
Solvent	31.8
RMS (bond lengths)	0.004
RMS (bond angles)	1.6
Favored (%)	90.6
Allowed (%)	8.8
Outliers (%)	0.1

1. Highest resolution shell is shown in parenthesis.
2. $R_{merge} = \Sigma |(I_i - \langle I_i \rangle | \Sigma I_i$ where I_i = intensity of the *i*th reflection and $\langle I_i \rangle$ = mean intensity.
3. R-factor = $\Sigma(|F_{obs}|\text{-}k|F_{calc}|)/ \Sigma |F_{obs}|$ and R-free is the R value for a test set of reflections consisting of a random 5% of the diffraction data not used in refinement.

Table S8. Data Collection and Refinement Statistics for *Mm*MgmA

	*Mm*MgmA Fe anomalous	*Mm*MgmA Native Dataset
**Data collection**		
Space group	$P\ 2_1 2_1 2_1$	$P\ 2_1 2_1 2_1$
Wavelength (Å)	1.73818	1.03316
Cell dimensions		
a, *b*, *c* (Å)	49.84, 60.89, 163.49	48.70, 72.14, 114.87
α, β, γ (°)	90, 90, 90	90, 90, 90
Resolution (Å)	50.0 – 2.65 (2.70 – 2.65)	44.9 – 2.08 (2.12 – 2.08)
No. of unique reflections	27027	22,172
R_{sym} or R_{merge}	0.094 (0.307)	0.078 (0.603)
R_{pim}	0.028 (0.092)	0.040 (0.322)
$I / \sigma I$	26.0 (9.4)	15.8 (2.2)
$CC_{1/2}$	1.007 (0.97)	0.997 (0.766)
Completeness (%)	96.5 (99.3)	89.2 (93.0)
Redundancy	12.5 (11.5)	3.7 (3.5)
**Refinement**		
Resolution (Å)		44.9 – 2.08 (2.12 – 2.08)
No. reflections		21368
R_{work} / R_{free}		0.2030 / 0.2531
No. atoms		3559
Protein		3301
Ligand/ion		101
Water		157
B-factors $Å^2$		
Protein		29.16
Ligand/ion		23.07
Water		29.07
R.m.s. deviations		
Bond lengths (Å)		0.002
Bond angles (°)		0.54
Clashscore		2.66
Ramachandran		
Most favored (%)		98.29
Allowed (%)		1.71
Outliers (%)		0
Number of TLS groups		4
PDB accession code		

Detailed Materials and methods

Bioinformatics methods. The genomic content of six methanogens were compared using the single gene phylogenetic profiling tool in the Department of Energy's Integrated Microbial Genomes (IMG) website (https://img.jgi.doe.gov/) using the default options. The putative functions of candidate genes were based on the IMG annotation and searches made with the Conserved Domain Search tool in NCBI (https://www.ncbi.nlm.nih.gov/Structure/cdd/wrpsb.cgi). Sequence similarity networks were generated using the Enzyme Function Initiative's Enzyme Similarity Tool (EFI-EST) using the *M. marburgensis* MgmA protein (Genbank accession number: WP_013296343) as a query. Isofunctional clusters were generated using an alignment score cutoff of 90, which was chosen to include the homologs of all strains for which the methylglutamine PTM has been experimentally validated. Genomic neighborhood networks were generated using an alignment score of 200 using the Enzyme Function Initiative's Genome Neighborhood Tool (EFI-GNT) with default settings. Sequence alignments and trees were generated using MUSCLE protein alignment and RAxML, respectively, which were executed within Geneious version 9.1.8. Maximum-likelihood trees were generated using RAxML version 7.2.8 using the substitution Matrix: BLOSUM62 using the MUSCLE alignment as an input. Branch support was calculated using the bootstrapping algorithm with 100 resamples. The tRNA modification enzyme MiaB from *Vibrio* species (Genbank accession number WP_045974662) was used an outgroup.

Molecular biology methods. Standard molecular biology methods were used throughout. Most plasmids were constructed using the HiFi assembly kit (New England Biolabs, Ipswich, MA, USA). Some plasmids were subsequently modified to enable the autonomous replication in *M. acetivorans* by in vitro cointegration with pAMG40 using BP clonase II (Thermo Fisher Scientific, Waltham, MA, USA) as previously described (1). Details of the plasmid constructions are provided in Table S2. Primers and synthetic DNA fragments used in these constructions are listed in Tables S3 & S4. CRISPR gene editing target sequences were found using the CRISPR site finder tool in Geneious and are listed in Table S5. All plasmids were verified by Sanger sequencing at the Roy J. Carver Biotechnology Center, University of Illinois at Urbana-Champaign. *Escherichia coli* WM4489 was used as the host strain for all plasmids generated in this study (4).

Construction and growth of microbial strains. Methods for growth and genetic manipulation of E. coli and M. acetivorans have been previously described (1, 5, 6). *M. acetivorans* strains generated in this project were verified by Sanger sequencing of PCR products containing the modified regions at the Roy J. Carver Biotechnology Center, University of Illinois at Urbana-Champaign.

Partial purification of MCR from *M. acetivorans* via ammonium sulfate precipitation and size-exclusion chromatography. Purification of untagged MCR used for mass spectrometric analyses of protein modification was performed under aerobic conditions using ammonium sulfate precipitation followed by size exclusion chromatography. In brief, appropriate strains were grown in 500 mL HS-medium with 50 mM TMA, 2 ug/mL puromycin, with 100 ug/mL tetracycline added as an inducer as needed. The cells were harvested by centrifugation (3,000 g) for 15 min at 4˚C. The cell pellet was losmotically lysed by resuspension in 10 ml of 100 mM Tris HCl (pH 8). The lysate was treated with DNase and cleared by centrifugation (17,000 g) for 30 min at 4˚ C. The lysate was then brought to 70% $(NH_4)_2SO_4$ saturation by addition of 23.3 mL of a saturated solution in 100 mM Tris HCl (pH 8) at 4˚ C. This solution was centrifuged at

95,834 g for 20 min at 4˚ C. After separating the supernatant, an additional 16.7 mL of the saturated solution was added to create an 80% saturated solution. The centrifugation step was repeated, and the resulting pellet containing MCR was resuspended in 300 uL of 100 mM Tris HCl (pH 8). The resuspended pellet was then passed through a 2 um filter, then loaded onto a HiLoad 16/60 Superdex 200 (Millipore Sigma). To identify which fraction contained MCR, 10 uL of each fraction was mixed with an equal volume of 2x Laemmli sample buffer (Bio-Rad) with 5% β-mercaptoethanol, boiled for 10 minutes, loaded on a 12% Mini-Protean TGX denaturing SDS-PAGE gel (Bio-Rad), and run at 220 V until the dye-front reached the bottom of the gel. The gel was stained with Gel Code Blue stain reagent (Thermo Fisher Scientific). Fractions containing MCR were then concentrated using Amicom Centrifugal filter Units (Millipore Sigma) with a 50 KDa cutoff.

Proteolytic digestion of purified MCR. 100 ug of purified MCR was digested with MS-grade trypsin (Thermo Fisher Scientific) at a 1:50 w/w ratio in 100 mM Tris HCl (pH 8) at 55° C for 1 hour. For digestion with GluC, 100 ug of MCR was digested with MS-grade GluC (Thermo Fisher Scientific) at a 1:50 w/w ratio in 500mM NH_4HCO_3 at 30° C for 30 minutes. The peptides from the digestions were desalted using StageTips (7) and the sample was subsequently lyophilized.

LCMS analyses of MCR peptide fragments. The lyophilized peptides were resuspended in 5% acetonitrile and 0.1% formic acid and approximately 1 ug was loaded onto a Solid Phase Extraction Micro Pillar Array C18 Reversed Phase column (Thermo Fisher Scientific) and a linear gradient of 2%-10% acetonitrile was used for 10 minutes. This gradient was followed by another gradient from 10%-36% acetonitrile over 90 minutes. Fractions were collected at 30 second intervals and infused directly into a Thermo Fisher Scientific Orbitrap Fusion ESI-MS for high-resolution electrospray ionization (ESI) MS/MS. The MS was operated using 120,000 resolution, 1 m/z isolation width (MS/MS), 50 normalized collision energy (MS/MS), 0.4 activation q value (MS/MS), and 30 ms activation time (MS/MS). Data analysis was performed using using the Qualbrowser application of Xcalibur software (Thermo Fisher Scientific) and the MASCOT server (Version 2.8.2).

Growth assays for *M. acetivorans* strains. *M. acetivorans* strains (WWM1184, WWM1187) were grown in single cell morphology in high salt medium (8, 9) containing one of the following: 125 mM methanol, 50 mM TMA or 40 mM sodium acetate. Prior to growth analyses, cells were adapted to each medium and temperature for a minimum of 5 generations, followed inoculation of the test medium using 3%(v/v) of a late-exponential phase culture. Growth rate was quantified by measuring the OD at a wavelength of 600 nm using a Spectronic 200E (Thermo Fisher Scientific) spectrophotometer. The mean of three independently calculated growth rates is reported.

Purification of MgmA from *M. acetivorans* for characterization and activity assays. TAP-tagged MgmA was purified from 10 L of a late-exponential phase culture of WWM1185 grown in HS media with 50 mM TMA, 2 ug/mL puromycin and 100 ug/uL tetracycline. Cell harvest and protein purification were performed as described (3) for MCR with the following changes: all purification and harvest steps were performed under anoxic conditions, the composition of the buffers are as follows, lysis buffer 50 mM HEPES, pH 7.5, 10 mM DTT, in addition the wash buffer was 250 mM KCl and the elution buffer had an additional 50 mM biotin.

Purification of MCR from *M. acetivorans* for crystallography. TAP-tagged MCR was purified as described (10) with the following modifications. The protein was purified under aerobic conditions from 2 L of late-exponential-phase culture of WWM1186 grown in HS + 50 mM TMA at 36˚C. The crystallography wash buffer is composed of 100 mM Tris.HCl and 300 mM NaCl (pH 8). The crystallography elution buffer contained 50 mM biotin in addition to the other components of the crystallography wash buffer. Four 1 mL fractions of purified MCR were collected and visualized using a 12% Mini-Protean TGX denaturing SDS-PAGE gel (Bio-Rad).

Cobamide extraction and analysis. Cobamide extraction and mass-spectrometric characterization was performed as previously described (11). To quantify the bound cobamide in MgmA a sample of the eluted protein was boiled 100 mM potassium cyanide for 5 minutes, followed by incubation under bright light for 15 minutes before centrifuging at 14000g for 5 minutes. The absorbance of the supernatant was measured at 367 nm and the extinction coefficient of 30800 was used to determine cyanocobalamin concentration.

MgmA activity assays. Reactions were performed in triplicate under strictly anoxic conditions at 25˚C. The reaction mixture was composed of 20 mM HEPES (pH7.5), 150 uM peptide substrate and 2 mM Ti(III) citrate, 7.5 uM MgmA. The reaction was initiated by addition of SAM to a final concentration of 500 uM. The reaction was quenched by adding an equal volume of 100 mM H_2SO_4, then analyzed via MALDI-TOF MS using alpha-cyano-4-hydroxycinnamic acid (CHCA) and matrix and a Bruker UltrafleXtreme mass spectrometer (Bruker Daltonics, Billerica, MA, USA) in reflector positive mode at the University of Illinois School of Chemical Sciences Mass Spectrometry Laboratory. Peptide concentrations were calculated by measuring the ratio between the modified and unmodified peptides within the same sample observed in the same acquisition(14).

Crystallization of *Ma*MCR Gln_{420} variant. The purified enzyme was concentrated to 25 mg/ml prior to crystallization via ultrafiltration. The crystallization trials utilized 2-µl sitting drops composed of 0.9:0.9:0.2 (protein:reservoir solution:additive screen) that were equilibrated against a 500-µl volume of the reservoir solution at room temperature. The reservoir solution contained 0.2 M ammonium acetate, 0.1 M sodium acetate (pH = 4), and 15-20% (w/v) PEG 4000. Prior to freezing by vitrification in liquid nitrogen, crystals were soaked in reservoir solution supplemented with an additional 20% (v/v) glycerol or 30% (w/v) PEG 4000. Diffraction data were collected at MacCHESS using an Dectris Eiger2 detector. Raw diffraction images were integrated and scaled using either XDS or AutoProc. Molecular replacement was carried out using the coordinates for the structure of wild-type *Ma*MCR. The initial model was subject to manual rebuilding, and cofactors were added to the model after the free R factor dropped below 0.30. Relevant crystallographic statistics are detailed in Table S7.

Plasmid construction of p*Mm*MgmA. The gene encoding *Methanothermobacter marburgensis* (gene *MTBMA_c15540,* UniProt ID: D9PY35_METTM) was optimized for expression in *E. coli* and ordered from Invitrogen GeneArt Gene synthesis with an added 5′ *Nde*I cut site and a 3′ *Xho*I cut site. The *Mm*MgmA gene was removed from the GeneArt pMA-T vector by digestion with the NdeI and XhoI restriction enzymes and subsequently ligated into linearized pET28a plasmid using T4 DNA ligase. The resulting plasmid was named p*Mm*MgmA. *E. coli* DH5α cells were transformed with p*Mm*MgmA, and the sequence was confirmed by DNA sequencing at Pennsylvania State Genomics Core Facility.

Overexpression and purification of *Mm*MgmA. Expression and purification of *Mm*MgmA was modified from previously established methods used to obtain soluble rSAM enzymes via heterologous expression in *E. coli* (15, 16). An *E. coli* BL-21(DE3) strain harboring the pDB1282 and pBAD42-BtuCEDFB plasmids was transformed with p*Mm*MgmA. A single colony of the resulting construct was used to inoculate 200 mL of an LB medium starter culture containing 50 µg/mL kanamycin, 50 µg/mL spectinomycin, and 100 µg/mL ampicillin. The starter culture was incubated overnight at 37° C and shaken at 250 rpm. A 4 mL aliquot of the starter culture was used to inoculate 4 L of ethanolamine minimal medium, containing equivalent antibiotic concentrations, in a non-baffled 6 L Erlenmeyer flask and grown at 37° C. At an OD_{600} = 0.3, arabinose was added to the culture to a final concentration of 0.2% (w/v) to induce expression of the *isc* and *btu* operons on the pDB1282 and pBAD42-BtuCEDFB, respectively. Simultaneously, 25 µM $FeCl_3$ was added to the medium as the iron source for FeS cluster biogenesis. The cultures were then grown to an OD_{600} = 0.6 and 50 µM IPTG was added to the growth to induce the expression of *Mm*MgmA. The temperature was reduced to 18 °C and the culture was incubated for 18 h before harvesting the cells by centrifugation at 7,000 x g. The harvested cells were flash-frozen and stored at -80° C until protein purification.

All remaining steps were performed in a Coy Laboratories anaerobic chamber or in an airtight vessel to ensure an oxygen-free environment. Cell paste was resuspended in lysis buffer (50 mM HEPES, pH 7.5, 300 mM KCl, 10% glycerol, 10 mM β-mercaptoethanol (BME), 4 mM imidazole) for 10 min. The following reagents and enzymes were added to the resulting solution and allowed to incubate for 5 min: 1 mg/mL Lysozyme, 0.1 mg/mL DNAse, 0.17 mg/mL PMSF, 0.8 µg/mL cysteine, 0.7 µg/mL $FeCl_3$, and 0.2% Triton x100. The cell suspension was then sonicated for a total of 5 min (45 seconds on, 7 min off) at 35% amplitude. The lysate was centrifuged at 45,000 x g and the resulting supernatant was loaded onto a Ni-NTA column equilibrated with ~100 mL of Lysis Buffer. The column was washed with 100 mL of lysis buffer to remove all non-His-tagged proteins. *Mm*MgmA was eluted from the column with 75 mL of elution buffer (50 mM HEPES pH 7.5, 300 mM KCl, 10% glycerol, 10 mM BME, 300 mM Imidazole). The eluate was concentrated in a 30 kDa MWCO Amicon Ultra Centrifugal Filter. *Mm*MgmA was buffer exchanged into storage buffer (50 mM HEPES pH 7.5, 300 mM KCl, 20% glycerol, and 1 mM DTT) via a PD-10 desalting column. The resulting protein is referred to as "As isolated *Mm*MgmA (AI *Mm*MgmA)." The cofactors of AI *Mm*MgmA were chemically reconstituted by incubating overnight in a reconstitution buffer (storage buffer plus 2.5 mM DTT, 150 µM $FeCl_3$, 150 µM Na2S, and 150 µM hydroxycobalamin) (16). The resulting protein mixture was further purified by size exclusion chromatography on a HiPrep 26/60 S200 column with an isocratic method using S200 buffer (50 mM HEPES pH 7.5, 300 mM KCl, 10% glycerol, and 10 mM DTT) as mobile phase. Fractions indicative of monomeric *Mm*MgmA were pooled, concentrated, and then buffer exchanged into storage buffer before flash-freezing and storage in liquid nitrogen (LN). The resulting protein is referred to as "Reconstituted *Mm*MgmA (RCN *Mm*MgmA)."

RCN *Mm*MgmA Activity Assays. Activity assays contained 20 µM RCN *Mm*MgmA, 200 µM peptide substrate, 500 µM SAM, 2 mM TiCitrate, 250 mM KCl, and 100 µM D-methionine-methyl-d_3 in 50 mM HEPES, pH 7.5. For LC-MS analysis, an aliquot of the reaction was quenched by a two-fold dilution in 100 mM sulfuric acid at each time point.

Quantification of 5′dAH, Methionine, and *S*-adenosylhomocysteine: Reaction aliquots were centrifuged at 13,100 g for 15 min at 4˚C to remove any precipitate. The supernatant was then injected into an Agilent Technologies 1290 Infinity II series UHPLC system coupled to a 6470

QQQ Agilent Jet Stream electrospray-ionization mass spectrometer. Analytes were chromatographically separated on an Agilent Zorbax Extend-C18 RRHD column (2.1 mm × 50 mm, 1.8 μm particle size) at 32.5˚C that was equilibrated in 95% solvent A (0.1% formic acid, pH 2.6) and 5% solvent B (acetonitrile). Throughout the duration of a single injection, the following gradient was applied: 0 to 0.5 min solvent B was held at 5%, 0.5 to 5 min solvent B increased from 5% to 35%, and from 5 to 6.5 min solvent B increased to 90%. Analytes were detected in positive mode using a multiple-reaction method. A standard curve of 5′dAH, Met, and SAH (500 nM through 300 μM) with 50 μM D-methionine-methyl-d_3 (internal standard) was prepared for quantification using the Agilent MassHunter Quantitative Analysis 10.1 Software.

***Mm*MgmA structure determination by X-ray crystallography.** X-ray diffraction datasets were collected at the General Medical Sciences and Cancer Institutes Collaborative Access Team (GM/CA-CAT) at the Advanced Photon Source, Argonne National Laboratory and the Berkeley Center for Structural Biology (BCSB) beamlines at the Advanced Light Source at Lawrence Berkeley National Laboratory. All datasets were processed using the HKL2000 or HKL3000 package, and structures were determined by single anomalous dispersion (SAD) phasing using Autosol/HySS or by molecular replacement using the program PHASER (17). Model building and refinement were performed with Coot and phenix.refine, respectively (18, 19). Ligand geometric restraints were obtained from the Grade Web Server (https://www.globalphasing.com.). Structures were validated and analyzed for Ramachandran outliers with the Molprobity server (20). Figures were prepared using PyMOL. Active site cavity mapping was prepared using Hollow (21).

Brown, plate-shaped RCN *Mm*MgmA crystals were generated via the hanging drop vapor diffusion method at room temperature by mixing 1 μL of a solution of *Mm*MgmA in storage buffer (5 mg/mL) with 1 μL of the well solution [0.2 M Calcium Chloride, 20% (*w/v*) PEG 3350, and 5 mM SAH]. Crystals were prepared for data collection by mounting on rayon loops followed by soaking in cryoprotectant solution [50% (*w/v*) PEG 3350] and flash-freezing in LN.

Diffraction datasets for single-wavelength anomalous diffraction (SAD) phasing were collected at the Fe *K*-edge x-ray absorption peak (1.73818 Å). A native dataset was collected on a separate crystal. Initial phasing attempts in phenix.autosol revealed that *Mm*MgmA likely contained a single iron-containing metallocofactors. The enhanced overall figure-of-merit (FOM) was 0.311 and the Bayes-CC was 38.82. Phenix.autobuild was used to generate an initial model of 366 residues out of 450 with an Rwork/Rfree of 0.28/0.33. The resulting model was then manually adjusted in Coot and refined in Phenix.This model was then used as the search model in phasing the native dataset by molecular replacement in Phenix. The final model consists of residues 3-35, 40-107, 110-150, 155-429, one [Fe_4S_4] clusters, and hydroxycobalamin. Data collection and refinement statistics are provided in Table XX.

Supplemental Material References

1. Guss AM, Rother M, Zhang JK, Kulkarni G, Metcalf WW. 2008. New methods for tightly regulated gene expression and highly efficient chromosomal integration of cloned genes for *Methanosarcina* species. Archaea 2:193.
2. Nayak DD, Metcalf WW. 2017. Cas9-mediated genome editing in the methanogenic archaeon *Methanosarcina acetivorans*. Proc Natl Acad Sci U S A 114:2976.
3. Nayak DD, Liu A, Agrawal N, Rodriguez-Carerro R, Dong SH, Mitchell DA, Nair SK, Metcalf WW. 2020. Functional interactions between posttranslationally modified amino acids of methyl-coenzyme M reductase in *Methanosarcina acetivorans*. PLoS Biol 18:e3000507.
4. Kim SY, Ju KS, Metcalf WW, Evans BS, Kuzuyama T, van der Donk WA. 2012. Different biosynthetic pathways to fosfomycin in *Pseudomonas syringae* and *Streptomyces* species. Antimicrob Agents Chemother 56:4175.
5. Metcalf WW, Zhang JK, Apolinario E, Sowers KR, Wolfe RS. 1997. A genetic system for Archaea of the genus *Methanosarcina*: liposome-mediated transformation and construction of shuttle vectors. Proc Natl Acad Sci U S A 94:2626.
6. Metcalf WW, Zhang JK, Wolfe RS. 1998. An anaerobic, intrachamber incubator for growth of *Methanosarcina spp*. on methanol-containing solid media. Appl Environ Microbiol 64:768.
7. Rappsilber J, Mann M, Ishihama Y. 2007. Protocol for micro-purification, enrichment, pre-fractionation and storage of peptides for proteomics using StageTips. Nat Protoc 2:1896-906.
8. Metcalf WW, Zhang JK, Shi X, Wolfe RS. 1996. Molecular, genetic, and biochemical characterization of the *serC* gene of *Methanosarcina barkeri* Fusaro. J Bacteriol 178:5797.
9. Sowers KR, Boone JE, Gunsalus RP. 1993. Disaggregation of *Methanosarcina spp*. and growth as single cells at elevated osmolarity. Appl Environ Microbiol 59:3832.
10. Nayak DD, Mahanta N, Mitchell DA, Metcalf WW. 2017. Post-translational thioamidation of methyl-coenzyme M reductase, a key enzyme in methanogenic and methanotrophic Archaea. Elife 6:e29218.
11. Fu H, Goettge MN, Metcalf WW. 2019. Biochemical characterization of the methylmercaptopropionate: cob(I)alamin methyltransferase from *Methanosarcina acetivorans*. J Bacteriol 201:e00130.
12. Backenköhler A, Eisenschmidt D, Schneegans N, Strieker M, Brandt W, Wittstock U. 2018. Iron is a centrally bound cofactor of specifier proteins involved in glucosinolate breakdown. Plos One 13:e0205755.
13. Rohaun SK, Imlay JA. 2022. The vulnerability of radical SAM enzymes to oxidants and soft metals. Redox Biol 57:102495.
14. Szájli E, Fehér T, Medzihradszky KF. 2008. Investigating the quantitative nature of MALDI-TOF MS. Mol Cell Proteomics 7:2410.
15. Lanz ND, Grove TL, Gogonea CB, Lee KH, Krebs C, Booker SJ. 2012. RlmN and AtsB as models for the overproduction and characterization of radical SAM proteins. Methods Enzymol 516:125.
16. Lanz ND, Blaszczyk AJ, McCarthy EL, Wang B, Wang RX, Jones BS, Booker SJ. 2018. Enhanced solubilization of class B radical S-adenosylmethionine methylases by improved cobalamin uptake in *Escherichia coli*. Biochemistry 57:1475.
17. Bunkóczi G, Echols N, McCoy AJ, Oeffner RD, Adams PD, Read RJ. 2013. Phaser.MRage: automated molecular replacement. Acta Crystallogr D Biol Crystallogr 69:2276.
18. Otwinowski Z, Minor W. 1997. Processing of X-ray diffraction data collected in oscillation mode. Methods Enzymol 276:307.

19. Adams PD, Afonine PV, Bunkóczi G, Chen VB, Davis IW, Echols N, Headd JJ, Hung LW, Kapral GJ, Grosse-Kunstleve RW, McCoy AJ, Moriarty NW, Oeffner R, Read RJ, Richardson DC, Richardson JS, Terwilliger TC, Zwart PH. 2010. PHENIX: a comprehensive Python-based system for macromolecular structure solution. Acta Crystallogr D Biol Crystallogr 66:213.
20. Williams CJ, Headd JJ, Moriarty NW, Prisant MG, Videau LL, Deis LN, Verma V, Keedy DA, Hintze BJ, Chen VB, Jain S, Lewis SM, Arendall WB, 3rd, Snoeyink J, Adams PD, Lovell SC, Richardson JS, Richardson DC. 2018. MolProbity: More and better reference data for improved all-atom structure validation. Protein Sci 27:293.
21. Ho BK, Gruswitz F. 2008. HOLLOW: generating accurate representations of channel and interior surfaces in molecular structures. BMC Struct Biol 8:49.
